# Celluloepidemiology—A paradigm for quantifying infectious disease dynamics on a population level

**DOI:** 10.1126/sciadv.adt2926

**Published:** 2025-05-16

**Authors:** My K. Ha, Anna Postovskaya, Maria Kuznetsova, Pieter Meysman, Vincent Van Deuren, Sabrina Van Ierssel, Hans De Reu, Jolien Schippers, Karin Peeters, Hajar Besbassi, Leo Heyndrickx, Betty Willems, Joachim Mariën, Esther Bartholomeus, Koen Vercauteren, Philippe Beutels, Pierre Van Damme, Eva Lion, Erika Vlieghe, Kris Laukens, Samuel Coenen, Reinout Naesens, Kevin K. Ariën, Benson Ogunjimi

**Affiliations:** ^1^ Center for Health Economics Research and Modelling Infectious Diseases (CHERMID), Vaccine and Infectious Disease Institute, University of Antwerp, Wilrijk, Belgium.; ^2^ Antwerp Center for Translational Immunology and Virology (ACTIV), Vaccine and Infectious Disease Institute, University of Antwerp, Wilrijk, Belgium.; ^3^ Antwerp Unit for Data Analysis and Computation in Immunology and Sequencing (AUDACIS), University of Antwerp, Antwerp, Belgium.; ^4^ ADReM Data Lab, Department of Mathematics and Computer Science, University of Antwerp, Antwerp, Belgium.; ^5^ Biomedical Informatics Research Network Antwerp (biomina), University of Antwerp, Antwerp, Belgium.; ^6^ Clinical Virology Unit, Department of Clinical Sciences, Institute of Tropical Medicine, Antwerp, Belgium.; ^7^ Department of General Internal Medicine, Infectious Disease and Tropical Medicine, Antwerp University Hospital, Edegem, Belgium.; ^8^ Laboratory of Experimental Hematology (LEH), Vaccine and Infectious Disease Institute, University of Antwerp, Wilrijk, Belgium.; ^9^ Flow Cytometry and Cell Sorting Core Facility (FACSUA), University of Antwerp, Wilrijk, Belgium.; ^10^ Virology Unit, Department of Biomedical Sciences, Institute of Tropical Medicine, Antwerp, Belgium.; ^11^ Department of Ecology and Evolutionary Biology, University of Antwerp, Antwerp, Belgium.; ^12^ The Virus Ecology Group, Institute of Tropical Medicine, Antwerp, Belgium.; ^13^ Centre for the Evaluation of Vaccination (CEV), Vaccine and Infectious Disease Institute, University of Antwerp, Wilrijk, Belgium.; ^14^ Global Health Institute, University of Antwerp, Wilrijk, Belgium.; ^15^ Laboratory of Medical Microbiology (LMM), Vaccine and Infectious Disease Institute (VAXINFECTIO), University of Antwerp, Wilrijk, Belgium.; ^16^ Center for General Practice, Department of Family Medicine and Population Health (FAMPOP), University of Antwerp, Wilrijk, Belgium.; ^17^ Department of Clinical Biology, Antwerp Hospital Network, Antwerp, Belgium.; ^18^ Department of Biomedical Sciences, University of Antwerp, Antwerp, Belgium.; ^19^ Department of Pediatrics, Antwerp University Hospital, Edegem, Belgium.

## Abstract

To complement serology as a tool in public health interventions, we introduced the “celluloepidemiology” paradigm where we leveraged pathogen-specific T cell responses at a population level to advance our epidemiological understanding of infectious diseases, using SARS-CoV-2 as a model. Applying flow cytometry and machine learning on data from more than 500 individuals, we showed that the number of T cells with positive expression of functional markers not only could distinguish patients who recovered from COVID-19 from controls and pre-COVID donors but also identify previously unrecognized asymptomatic patients from mild, moderate, and severe recovered patients. The celluloepidemiology approach was uniquely capable to differentiate health care worker groups with different SARS-CoV-2 exposures from each other. T cell receptor (TCR) profiling strengthened our analysis by revealing that SARS-CoV-2–specific TCRs were more abundant in patients than in controls. We believe that adding data on T cell reactivity will complement serology and augment the value of infection morbidity modeling for populations.

## Teaser

Celluloepidemiology offers cell-driven insights into human immunity against pathogens on a population level.

## INTRODUCTION

Serology has been a powerful tool in public health, infectious disease research, and the evaluation of vaccination programs (*1*). In seroepidemiology, data on antibody prevalence and titers in (human) serum are collected and analyzed to examine the distribution and determinants of infections in populations (*2*). The emergence of severe acute respiratory syndrome coronavirus 2 (SARS-CoV-2) at the end of 2019 has caused a global pandemic of COVID-19 and, thus, required population-wide epidemiological studies to investigate the causes, risk factors, outcomes, and eventually relieve the associated burden. However, the serological analysis of patients with COVID-19 is complicated by the variability of the SARS-CoV-2–induced antibody responses. For example, SARS-CoV-2 antibody titers are higher in patients with severe disease and lower in young, asymptomatic, or pauci-symptomatic individuals (*3*, *4*). Although immunoglobulin G (IgG) antibodies targeting SARS-CoV-2 receptor binding domain (RBD) were reported to persist at detectable levels beyond 3 months after symptom onset (*5*, *6*), IgM and IgA responses were short-lived and waned within 2.5 months, especially in asymptomatic cases (*3*, *5*, *7*).

On the other hand, SARS-CoV-2–induced cell-mediated immunity is considered more sustained but remains poorly characterized. Within cell-mediated immunity, the role of T cells in the exacerbation of COVID-19 and their potential to provide long-term immunity against SARS-CoV-2 has been increasingly discussed and studied. There is evidence that, compared with that from healthy controls, T cell compartment from patients with COVID-19 displays several alterations in the proliferation, expression of lineage-specifying receptors, and production of cytokines. For example, increases in the expression of inhibitory receptors such as programmed cell death protein 1 (PD-1), T cell immunoglobulin and mucin domain-containing protein 3 (TIM-3), lymphocyte activation gene 3 (LAG-3), and cytotoxic T-lymphocyte-associated protein 4 (CTLA-4) have been observed in highly activated or possibly exhausted T cells in severe acute COVID-19 cases (*8*–*12*). An increase in CD38^+^HLA-DR^+^ activated CD8^+^ T cell population was also reported in many patients with active SARS-CoV-2 infection (*8*, *9*, *13*). T cell activation in patients with acute COVID-19 was found to be skewed toward a T helper 17 (T_H_17) functional phenotype, which implies a potential role of T_H_17-mediated immunopathology in COVID-19 (*14*). Regulatory T cells and ICOS^+^CD38^+^ circulating follicular helper T cells have also been reported to be altered in patients with COVID-19 (*9*, *15*). CD4^+^ T cells in patients who recovered from mild COVID-19 were found to gain a typical memory phenotype with high expression of IL-7Rα (*16*). Other studies have targeted the functions of T cells in recognizing and acting against the structural SARS-CoV-2 viral proteins spike, membrane, and nucleocapsid (NP) (*17*–*20*). T cell receptor (TCR) sequencing (TCR-seq) has also shown that T cells can offer high sensitivity and specificity in the detection of past SARS-CoV-2 infection (*21*, *22*). Therefore, deeper and more comprehensive immune profiling of antigen-specific TCRs could enable better discrimination between infected and uninfected individuals, as well as improve our understanding of cell-mediated immunity against pathogens and its potential relevance in inferring susceptibility differences in the population. We believe that the systematic study of T cell responses against pathogens on a population level could introduce an unprecedented paradigm referred to as “celluloepidemiology.” The celluloepidemiology approach could leverage the assessment of human immune response dynamics against various pathogenic agents, given the diversity of biomarkers expressed and secreted by antigen-specific T cells in contrast to the unidimensional nature of antibodies (*23*).

In this study, we present a feasible experimental setup and the essential complementary computational approaches to perform celluloepidemiology on a SARS-CoV-2 model. We will compare celluloepidemiology with seroepidemiology at four levels: (i) sensitivity (i.e., the capacity to measure the genuine proportion of previously infected individuals), (ii) specificity (i.e., the capacity to identify individuals who have not been infected), (iii) morbidity/disease categorization (i.e., the capacity to distinguish between different disease groups among infected individuals), and (iv) health-related consequences [i.e., the capacity to detect immune alterations after an infection, such as in long Covid cases; (*24*)].

## RESULTS

### Comparison of ex vivo immunological phenotypes between patients who recovered from COVID-19, household members, controls, and pre-COVID donors

To obtain the best T cell surface markers that would then allow us to look into antigen-specific T cell responses at a population level, we analyzed peripheral blood mononuclear cells (PBMCs) of 30 patients who recovered from COVID-19 and 15 pre-COVID blood donors stimulated by SARS-CoV-2–specific major histocompatibility complex (MHC) class I– and class II–specific peptide pools (thus, without expected cross-reactivity with other coronaviruses), as well as SARS-CoV-2 membrane protein peptide pools (SC2-MP) using an extensive 33-isotope mass cytometry [cytometry by time-of-flight (CyTOF)] panel (table S1) and applied the Boruta algorithm (*25*) to look for the best differentiation between these groups. Figure S1 displays the selected T cell surface markers. High-importance values indicate markers whose expressions differ greatly between controls, patients, and patient subgroups (i.e., mild, moderate, and severe COVID-19). The Boruta-selected membrane markers were CTLA-4, human leukocyte antigen (HLA-DR), PD-1, TIM-3, CD27, CD28, CD38, Fas, T cell immunoreceptor with Ig and ITIM domains (TIGIT), CD154, OX40, CD137, and CD69 (fig. S1). These markers were divided into two panels: The first panel, taking into account representative markers of cells’ lineage and functional status, consisted of CD45RA, CCR7, CD27, CD28, CD38, Fas, TIGIT, CD154, OX40, CD137, and CD69 among other markers; and the second panel included the remaining CTLA-4, HLA-DR, PD-1, and TIM-3.

In a second phase, we tried to assess whether previous SARS-CoV-2 infection would continue to leave an impression on the immune system after more than 3 months. To achieve this, we analyzed the PBMCs of 478 individuals, including 166 patients who recovered from COVID-19, 29 household members of patients who recovered from COVID-19, 259 controls, and 24 pre-COVID donors (group definitions are presented in Methods). Household members [i.e., individuals who lived in the same household with patients with COVID-19 and thus had close contact and high chance of exposure to SARS-CoV-2 but did not have confirmed infection, either by polymerase chain reaction (PCR) or IgG test] were involved in this analysis to examine the effects of SARS-CoV-2 exposure on T cells after more than 3 months. The PBMCs were stimulated by MHC class I and class II SARS-CoV-2–specific and SC2-MP. Flow cytometry measurements were performed using the first panel (table S2) to broadly examine the immunophenotype profiles of different groups.

First, FlowSOM was implemented to semiautomatically gate CD4^+^ and CD8^+^ T cell subsets based on CD45RA and CCR7 expressions (fig. S2). Differences in T cell frequencies between different groups indicate that the naïve T cell subset was reduced in patients who recovered from COVID-19, the effector memory subset was increase in patients who recovered from COVID-19 and household members, whereas the central memory and terminally differentiated effector memory subsets were more present in patients who recovered from severe COVID-19 than in other groups. It is, however, worth noting that the distribution of memory and naïve T cell compartments is strongly affected by age. In our cohort, severe COVID-19 cases were all older than 57 years at the time of recruitment, whereas other groups included younger donors (table S5). In fig. S1, we performed statistical significance test on the age of different groups and showed that median age of severe COVID-19 cases was significantly higher than that of other groups. However, this does not disregard our observation of CD4^+^ and CD8^+^ naïve and memory compartments but rather adds to the epidemiological perspective of this study.

### Patients who recovered from COVID-19 had more SARS-CoV-2–specific T cells than controls

**Fig. 1.**
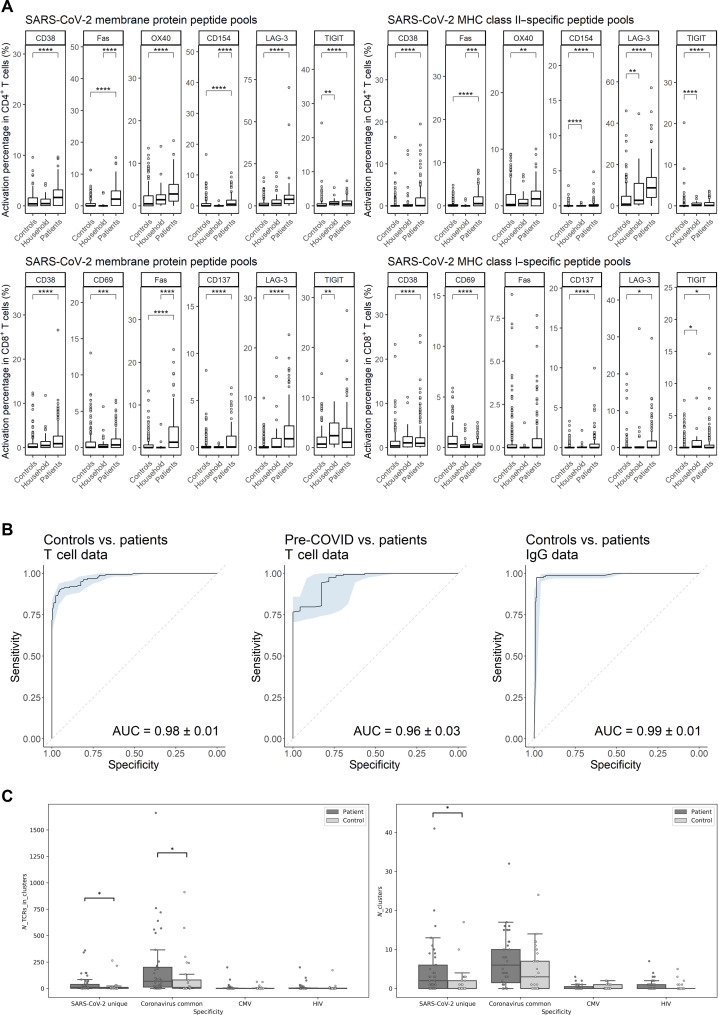
Comparison of SARS-CoV-2–specific T cell immune response between controls, patients, and household members. (**A**) Net percentages of T cells with positive expression of functional markers out of the total numbers of CD4^+^ and CD8^+^ T cells poststimulation (normalized with unstimulated controls). Horizontal lines represent median values of each group. Statistical significance was analyzed by Kruskal-Wallis test with Bonferroni correction. (**B**) Receiver operating characteristics (ROCs) and areas under the curve (AUCs) from leave-one-out cross-validation of random forest classifiers using T cell data and IgG data. (**C**) Numbers of clustered TCRs and clusters specific to SARS-CoV-2, coronaviruses, cytomegalovirus (CMV), and HIV in patients and controls. Statistical significance was analyzed by nonparametric *t* test with Benjamini-Hochberg correction. *P ≤ 0.05; **P ≤ 0.01; ***P ≤ 0.001; ****P ≤ 0.0001.

To explore further the complementary values of celluloepidemiology to seroepidemiology, we compared SARS-CoV-2–specific IgG and T cell data between patients who recovered from COVID-19 and controls. Figure 1A displays that, after stimulation with SC2-MP, CD4^+^ and CD8^+^ T cells in recovered patients expressed significantly higher levels of CD38, Fas, TIGIT, CD154, OX40, CD137, and CD69 (*P* ≤ 0.0001) than those in controls, emphasizing that there is a great number of T cells getting activated, exhausted, or apoptotic upon reencounter with SARS-CoV-2 peptides in recovered patients even more than 3 months after COVID-19 onset. Household members had lower Fas expression than patients (*P* ≤ 0.0001) but higher TIGIT expression than controls (*P* ≤ 0.01), indicating non-apoptotic exhaustion of T cells following reencounter with SARS-CoV-2 ex vivo. In terms of serology, fig. S3A reveals that recovered patients had significantly higher levels of SARS-CoV-2 NP, RBD, and S1-S2–specific IgG (*P* ≤ 0.0001) than controls. Household member had higher IgG reactivity than controls (*P* ≤ 0.01) but lower than patients (*P* ≤ 0.01). Simultaneously, we developed T cell classifiers that would allow us to adequately differentiate patients from controls. We applied random forest algorithm on the flow cytometry data. Expressions of eight functional markers (i.e., CD38, CD69, Fas, OX40, CD137, CD154, LAG-3, and TIGIT) in eight T cell populations were used as parameters on which the random forest classifiers were developed. Leave-one-out cross-validation of the first bilateral classifiers confirms that patients could be differentiated from controls and pre-COVID donors using flow cytometry T cell data with area under the curve (AUC) = 0.98 ± 0.01 and 0.96 ± 0.03, which was comparable to the classifier between controls and patients using IgG data with AUC = 0.99 ± 0.01 (Fig. 1B). On the basis of the results of the first T cell classifiers, a separate group of individuals were defined as “asymptomatic patients” as they initially were recruited to be control cases who did not report COVID-19–related symptoms nor knowingly exposure but were classified as “patients” by the T cell classifier. We note that the celluloepidemiology framework allowed this discovery of asymptomatic patients.

After looking into SARS-CoV-2–specific T cells on a phenotypic level, we took a step further by investigating the ex vivo CD8^+^ TCR repertoires. Because similar TCRs that cluster together generally target the same epitope and, thus, could reveal traces of T cell response, we compared the prevalence of CD8^+^ TCR clusters between controls and patients (Fig. 1C). Regarding the number of unique TCRs in the respective repertoires, recovered patients had significantly more CD8^+^ clusters than controls (*P* ≤ 0.001). We further differentiated the CD8^+^ TCRs between those uniquely reactive to SARS-CoV-2 and those that were considered cross-reactive to different coronaviruses. Figure 1C reveals that there was a significantly higher number of SARS-CoV-2 unique clusters and TCRs per cluster in recovered patients compared to healthy controls (*P* ≤ 0.05). Patients were also found to have more coronavirus-specific TCRs per cluster than healthy controls (*P* ≤ 0.05). Cytomegalovirus and HIV were included as negative controls. Table S8 contains a detailed list of annotated TCR-epitope specificity.

### T cell immune profiling is comparable to serological testing in identifying patients who recovered from mild, moderate, and severe COVID-19

**Fig. 2.**
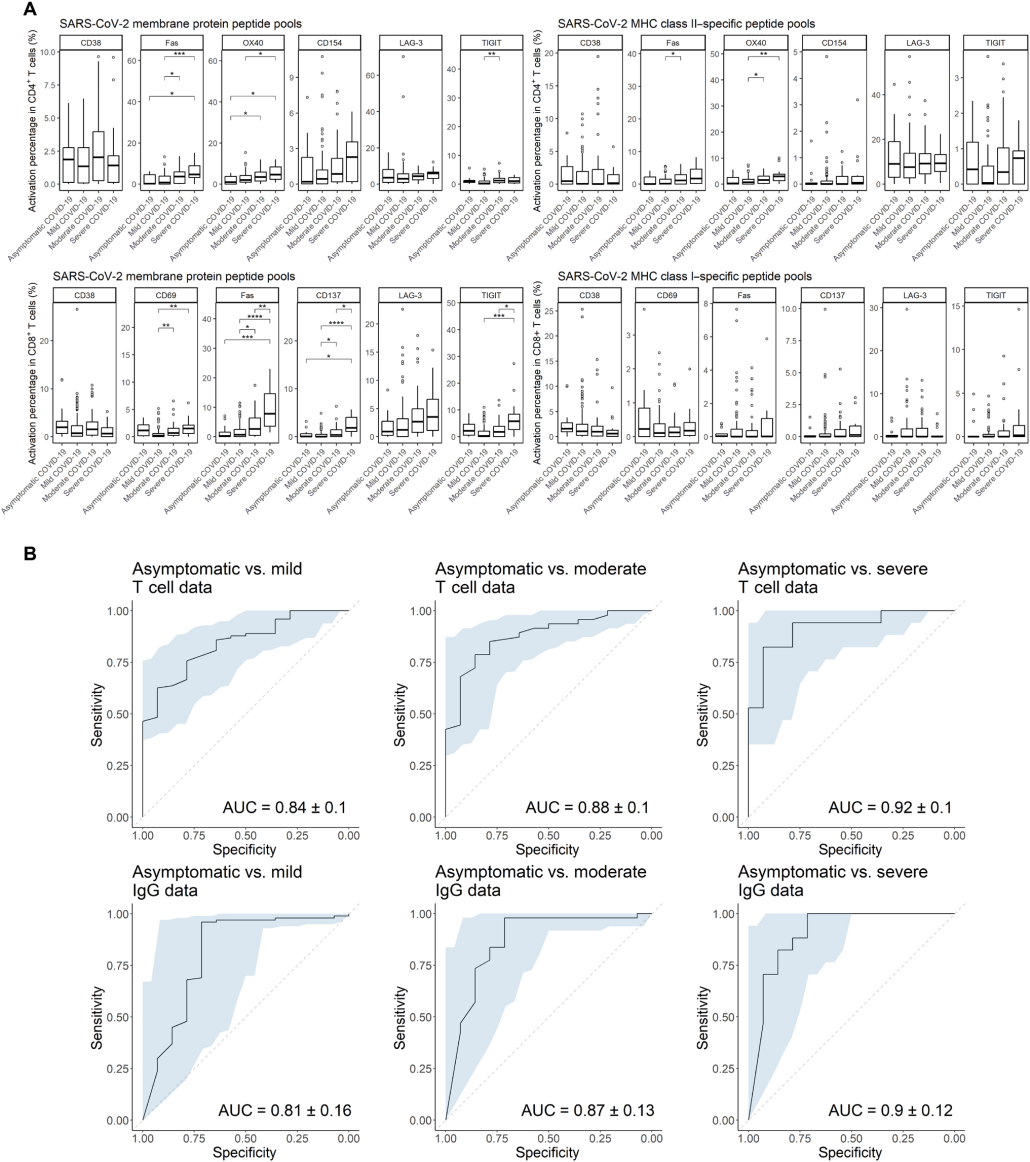
Comparison between asymptomatic, mild, moderate, and severe patients. (**A**) Net percentages of T cells with positive expression of functional markers out of the total numbers of CD4^+^ and CD8^+^ T cells poststimulation (normalized with unstimulated controls). Horizontal lines represent median values of each group. Statistical significance was analyzed by Kruskal-Wallis test with Bonferroni correction. *P ≤ 0.05; **P ≤ 0.01; ***P ≤ 0.001; ****P ≤ 0.0001. (**B**) ROCs and AUCs from leave-one-out cross-validation of random forest classifiers using flow cytometry T cell data and IgG data.

Next, we investigated whether celluloepidemiology and/or seroepidemiology could differentiate between previous mild, moderate, and severe COVID-19. Asymptomatic and mild patients were effectively distinguished from moderate and severe patients using both IgG and T cell data (Fig. 2A and fig. S3B). Looking at serological data, we found that the amount of SARS-CoV-2 RBD, NP, and S1-S2 antigen–specific IgG significantly increased as COVID-19 severity increased (*P* ≤ 0.01). However, moderate and severe patients could only be distinguished from each other by RBD reactivity (*P* ≤ 0.05), not by NP and S1-S2 reactivity. Looking at flow cytometry data, we observed that, upon stimulation by SC2-MP, CD4^+^ T cells displayed an increase in Fas and OX40 expressions as COVID-19 severity increases from asymptomatic to severe (*P* ≤ 0.05). A similar pattern could be observed in CD8^+^ T cells upon the same stimulation in the expressions of CD69, Fas, CD137, and TIGIT (*P* ≤ 0.05). Particularly, moderate and severe patients differed significantly in their Fas, CD137, and TIGIT expressions (*P* ≤ 0.05). These observations are consistent with the increased expression of TIGIT and Fas in severe COVID-19 cases reported by Neidleman *et al*. (*26*). The correlation between COVID-19 severity and T cells’ expression of CD137, Fas, OX40, and TIGIT reflects an increase in T cell differentiation from naïve to effector/memory phenotypes upon SARS-CoV-2 reencounter as severity increases. This finding adds an important insight, on a population level, to the understanding of pathogen-specific immunity postinfection that could not be obtained from serology. The multilateral T cell classifiers also performed as effectively as the serology-based approach (Fig. 2B). Using T cell flow cytometry data, asymptomatic patients could be better distinguished from mild, moderate, and severe COVID-19 with AUCs of 0.84 ± 0.10, 0.88 ± 0.10, and 0.92 ± 0.10, respectively, compared to the other pairs (i.e., mild versus moderate, mild versus severe, and moderate versus severe). This is comparable to their equivalents using IgG data, with AUCs being 0.81 ± 0.16, 0.87 ± 0.13, and 0.90 ± 0.12. Overall, these results indicate that a T cell–based infection classification on a population level would yield similar accuracy to the serology-based approaches. However, it should be noted that, in this study, we considerably augmented the capacity of serology to classify and stratify by applying our machine learning framework.

### In-depth multiparameter characterization of SARS-CoV-2–specific T cell responses using FlowSOM clustering

**Fig. 3.**
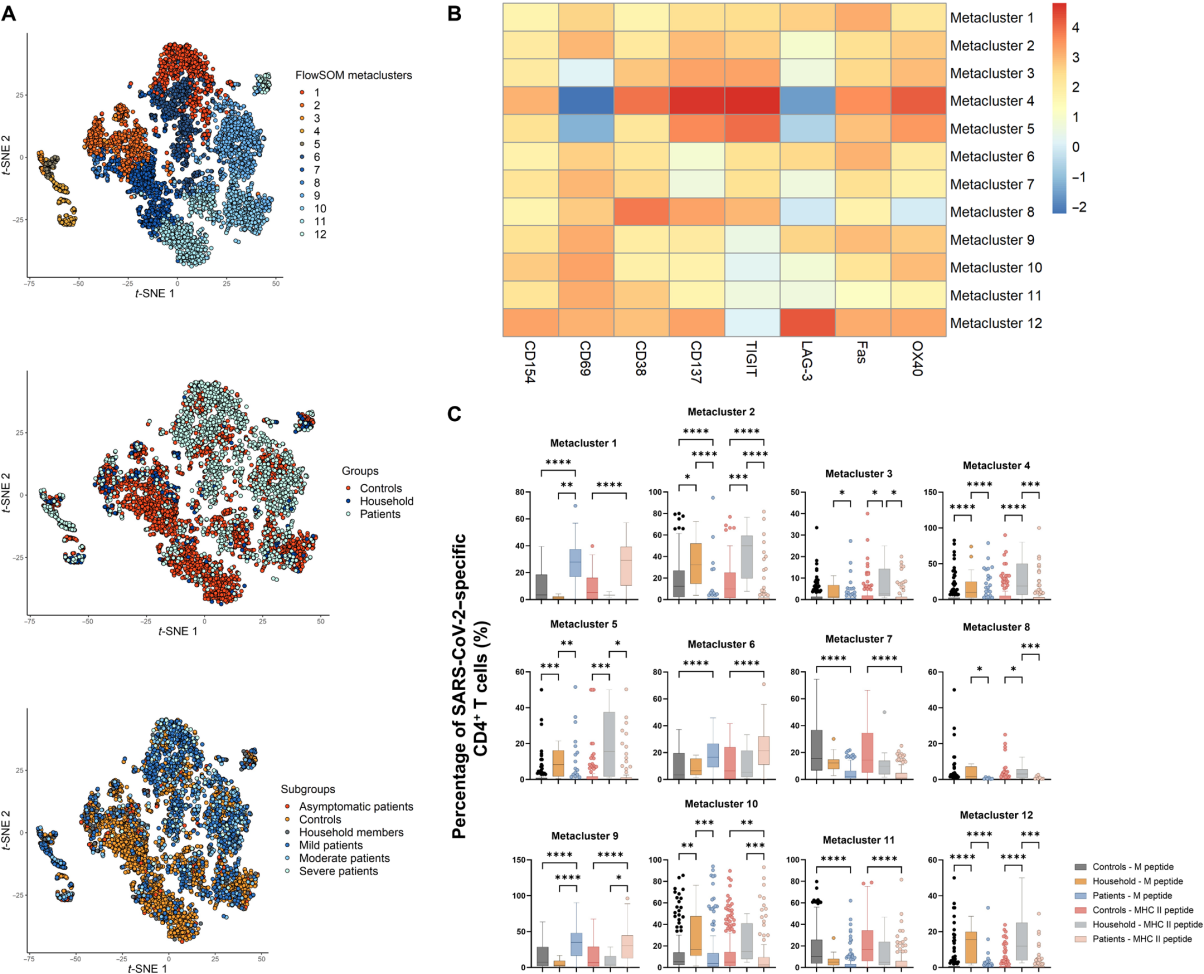
FlowSOM clustering applied on CD4^+^ T cells of controls, patients, and household members. (**A**) *t*-SNE representation of 12 metaclusters projected by cohort groups and subgroups. (**B**) Mean fluorescence intensity of individual activation marker in each metacluster. (**C**) Percentage of SARS-CoV-2–specific CD4^+^ T cells in each metacluster. Horizontal lines represent median values of each group. Statistical significance was analyzed by Kruskal-Wallis test with Dunn’s correction. *P ≤ 0.05; **P ≤ 0.01; ***P ≤ 0.001; ****P ≤ 0.0001. In agreement with the uniparameter approach (Fig. 1A). (B) and (C) revealed that CD4^+^ T cells in patients had high Fas expression (corresponding to metaclusters 1, 6, and 9), while CD4^+^ T cells in household members overexpressed CD154, OX40, CD38, Fas, and TIGIT (corresponding to metaclusters 2, 4, 5, 10, and 12).

**Fig. 4.**
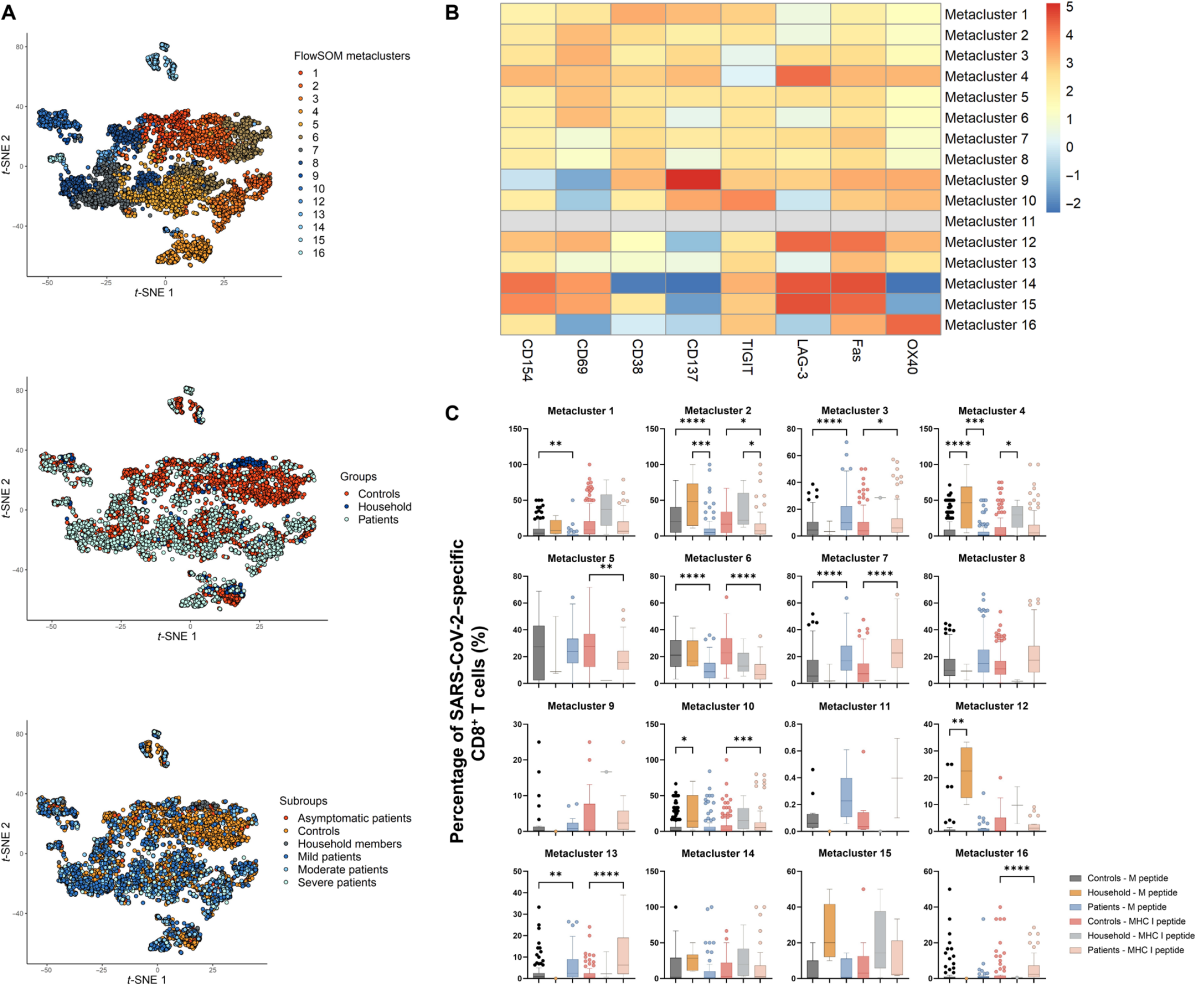
FlowSOM clustering applied on CD8^+^ T cells of controls, patients, and household members. (**A**) *t*-SNE representation of 16 metaclusters projected by cohort groups and subgroups. (**B**) Mean fluorescence intensity of individual activation marker in each metacluster. (**C**) Percentage of SARS-CoV-2–specific CD8^+^ T cells in each metacluster. Horizontal lines represent median values of each group. Statistical significance was analyzed by Kruskal-Wallis test with Dunn’s correction. *P ≤ 0.05; **P ≤ 0.01; ***P ≤ 0.001; ****P ≤ 0.0001. In agreement with the uniparameter approach (Fig. 1A). (B) and (C) highlighted that CD8^+^ T cells in patients had elevated levels of Fas and CD69 (i.e., metaclusters 3, 7, and 13), while CD8^+^ T cells in household members overexpressed CD137, CD69, LAG-3, Fas, and TIGIT (i.e., metaclusters 4, 10, and 12).

One of the potential advantages of the celluloepidemiology approach would be the inherent multiparameter capacity imbedded in T cell assays compared to the limited parameters (at best IgG and IgM titers, and perhaps neutralization assays and avidity testing) in serological assays. To explore this further, CD4^+^ and CD8^+^ T cells in controls, patients who recovered from COVID-19, and household members were gated by their expressions of eight functional markers (i.e., CD154, OX40, CD137, CD69, CD38, Fas, LAG-3, and TIGIT) after stimulation with SARS-CoV-2 peptides. Cells having higher expression of more than two activation markers after stimulation with SARS-CoV-2–derived peptides compared to unstimulated controls were here defined as SARS-CoV-2–specific T cells. To gain more insights into these SARS-CoV-2–specific T cells, we applied FlowSOM clustering in comparing expression of eight activation markers and identified 12 metaclusters for CD4^+^ T cells (Fig. 3) as well as 16 metaclusters for CD8^+^ T cells (Fig. 4). *t*-distributed stochastic neighbor embedding (*t*-SNE) representation of the data highlighted a distinction between controls and patients, but not so much between asymptomatic patients and patients with mild, moderate, and severe COVID-19 (Figs. 3A and 4A).

We looked further into SARS-CoV-2–specific T cell immune responses in controls, patients, and household members by investigating the expressions of PD-1, HLA-DR, TIM-3, and CTLA-4 in SARS-CoV-2–specific CD154^high^OX40^high^ CD4^+^ and CD137^high^CD69^high^ CD8^+^ T cells (figs. S4 and S5). In CD154^high^OX40^high^ CD4^+^ T cells (fig. S4), PD-1 expression was significantly higher in patients than in household members (*P* ≤ 0.05). In CD137^high^CD69^high^ CD8^+^ T cells (fig. S5), we found CTLA-4, HLA-DR, and PD-1 significantly higher in patients than in controls (*P* ≤ 0.01), while TIM-3 was highly expressed in patients and household members compared to controls (*P* ≤ 0.05). It is evident that CD8^+^ T cells in patients who recovered from COVID-19 and their household members (who were exposed to SARS-CoV-2) became more activated and exhausted than CD4^+^ T cells after reexposure ex vivo.

### Celluloepidemiology allows differentiation between health care worker groups

**Fig. 5.**
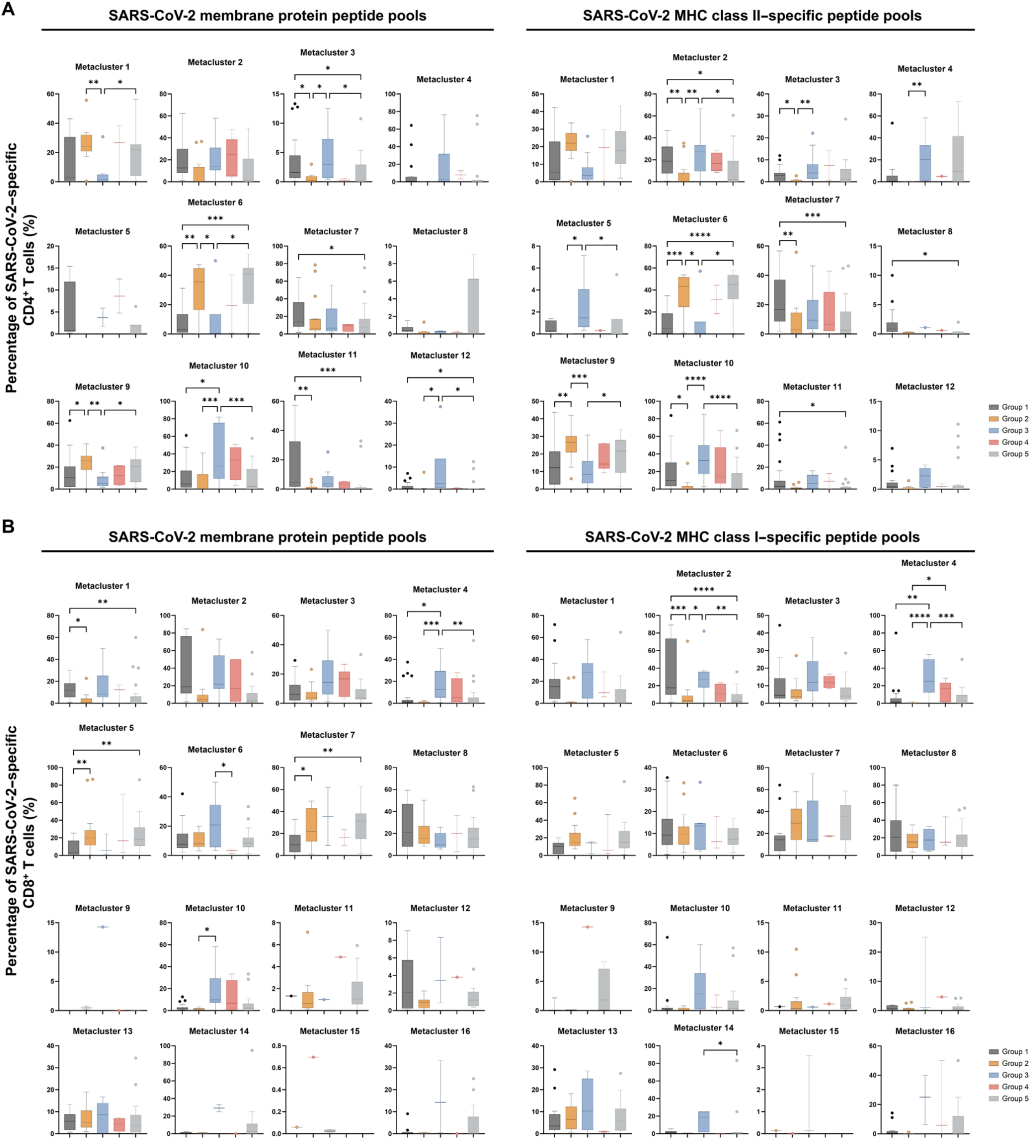
Percentage of SARS-CoV-2–specific T cells in health care workers in each metacluster of the FlowSOM model developed based on controls’, patients’, and household members’ data. (**A**) CD4^+^ T cells based on the FlowSOM model in Fig. 3. (**B**) CD8^+^ T cells based on the FlowSOM model in Fig. 4. Horizontal lines represent median values of each group. Statistical significance was analyzed by Kruskal-Wallis test with Dunn’s correction. *P ≤ 0.05; **P ≤ 0.01; ***P ≤ 0.001; ****P ≤ 0.0001.

Beside the control, recovered patient, and household member groups, the health care worker cohort could also offer interesting and relevant insights due to their frequent exposure to SARS-CoV-2 in a professional context. To explore this scenario, we recruited different health care worker groups (hospital personnel and general practitioners having worked for minimal 4 weeks on COVID-19 wards more than 3 months before sampling) who were very likely exposed to SARS-CoV-2 before recruitment (group 1: PCR negative or not tested, IgG negative, without fever; group 2: PCR negative or not tested, IgG negative, with fever; group 3: PCR negative or not tested, IgG positive, without fever; group 4: PCR negative or not tested, IgG positive, with fever; and group 5: PCR positive). Groups 1 and 2 (both having negative PCR and serology tests) were not distinguishable from each other by their SARS-CoV-2 RBD, NP, and S1-S2–specific IgG reactivity (fig. S9) but showed substantial differences in their T cell metacluster percentages (Fig. 5). It was evident in Fig. 5A that, among SARS-CoV-2–specific CD4^+^ T cells, group 1 had a higher percentage of metacluster 3 than group 2 (*P* ≤ 0.05), which represented a high expression of TIGIT and OX40. In contrast, group 2 had higher percentages of metaclusters 6 and 9 than group 1 (*P* ≤ 0.05), which highlights the difference in Fas expression between these two groups. Figure 5B reveals that, among SARS-CoV-2–specific CD8^+^ T cells, group 1 surpassed group 2 in the percentages of metacluster 1 (*P* ≤ 0.05, stimulated with membrane peptide pools) and metacluster 2 (*P* ≤ 0.001, stimulated with MHC class I peptide pools), which represented cells with high expression of CD69, CD38, and CD137. In contrast, group 2 surpassed group 1 in the percentages of metaclusters 5 and 7 (*P* ≤ 0.05), which contained cells with elevated levels of CD69 and Fas. This highlights that, despite having no confirmed SARS-CoV-2–positive tests (PCR and/or serology), health care providers in groups 1 and 2 had CD4^+^ and CD8^+^ T cells that showed signs of previous SARS-CoV-2 encounters, which was only reflected in their T cell signatures but not in their IgG reactivity. T cells of those in group 1 displayed SARS-CoV-2–specific activation and exhaustion, whereas T cells of those in group 2 displayed apoptosis susceptibility. Group 5, despite having tested positive by PCR, could not be distinguished from groups 3 and 4 (both having PCR negative or not taken) on the basis of their IgG data, but they were differentiated on the basis of Fas and CD137 expression in CD8^+^ T cells. Celluloepidemiology thus offers a unique approach to distinguish frequently exposed individuals from each other, which would not have been possible if only serology was used.

### Patiens with long Covid displayed elevated humoral and cellular immune responses against SARS-CoV-2

Following recovery, several patients reported suffering from the post-acute sequelae of COVID-19, also referred to as “long Covid,” which concerns a plethora of persistent symptoms (*27*, *28*). In our current data, there were 79 (of 166) recovered patients with long Covid (potentially indicating a recruitment bias), 40 of which were initially classified as mildly ill patients during the acute phase, 28 were moderately ill, and 11 were severely ill (table S4). Their long Covid symptoms commonly included fatigue, shortness of breath, persistent loss of taste and smell, muscle ache, and chest pressure. Some symptoms were relatively peculiar, such as liver inflammation, diminished eyesight, reduced or altered hearing, hormonal unbalance, cognitive dysfunction (including memory decline and concentration difficulties), palpitations, and limited lung capacity (one severe patient was listed for double lung transplant). These patients with long Covid had higher IgG reactivity to RBD, NP, and S1-S2 antigens and more up-regulated expression of Fas, OX40, CD154, LAG-3, TIGIT in CD4^+^ T cells and CD69, Fas, CD137, LAG-3 in CD8^+^ T cells compared to uninfected controls, asymptomatic patients, and patients with mild and moderate COVID-19 (figs. S6 and S7).

## DISCUSSION

In classical epidemiology, the incidence, prevalence, and distribution of infectious diseases are analyzed at a population level. It offers data to study the effectiveness of intervention strategies such as lockdowns, vaccination, and screening. One of the most common methods to estimate these quantities uses the serological footprint of an infection on the host. Seroepidemiological studies document the presence and kinetics of infection- or vaccine-induced antibodies. Studying of serological profiles in a population as a function of age and time and comparing these profiles between different regions have delivered important insights into the infectivity of pathogens, the identification of risk groups, the transmission routes of infections, the impact of immunization programs, and basic pathophysiological mechanisms. Although seroepidemiology has been a key tool to define successful public health interventions worldwide, it is fundamentally limited due to its use of antibodies as the sole marker of infectious disease immunity.

With the concept of celluloepidemiology, we would like to introduce an approach of studying T cell response against pathogens on a population level, thereby allowing more profound insights when analyzing immune responses from large (potentially not annotated) study groups. We consider celluloepidemiology as an added value to seroepidemiology thanks to the existence of T cell phenotypes and the expression of a diversity of proteins detectable on the T cell surface, thus allowing multidimensional T cell characterization. In addition, the extremely broad repertoire of TCRs, which can simultaneously recognize and memorize distinct pathogenic antigens, has recently been proven to be highly sensitive and specific in the detection of past SARS-CoV-2 infection (*29*). This implies that for large-scale modeling of infection morbidity, adding data on T cell reactivity will complement serology and augment the value of the models.

Another limitation of conventional seroepidemiology is that antibody titers declined over time after COVID-19 clearance (*30*). Cellular immunity against SARS-CoV-2, however, was found to decay more slowly over time than neutralizing antibody titers (*31*). A report on patients who had recovered from SARS-CoV-1 indicated that cellular immune responses were maintained for nearly two decades, whereas memory B cells and antibody responses could not be detected in most individuals at that point (*32*). Studies have shown that SARS-CoV-2–specific cellular immune responses remained detectable in recovered patients 8 months after infection, although higher in CD4^+^ T cells than in CD8^+^ T cells (*33*), which agrees with our data. Cohen *et al.* (*34*) observed that SARS-CoV-2–specific antibody responses and T cell activities might persist for 8 months following infection, which also confirms our findings.

Here, we applied the proposed celluloepidemiology paradigm to characterize SARS-CoV-2–specific T cell responses in patients recovered from COVID-19 for over 3 months and compared them to unexposed controls, household members of the patients who recovered from COVID-19, general practitioners, hospital health care workers, and pre-COVID controls. Using experimental and computational approaches, we showed that the number of CD4^+^ and CD8^+^ T cells with positive expression of functional markers, after stimulation with SARS-CoV-2 proteins, could be used to distinguish patients who recovered from COVID-19 from controls and pre-COVID donors, as well as differentiate asymptomatic patients from mild, moderate, and severe patients. To achieve this, we used automated T cell clustering algorithms like FlowSOM (*35*) and random forest (*36*) to maximize output from our data. Applying machine learning algorithms on three-dimensional Luminex-based serological data allowed previous SARS-CoV-2 infection classification with more than 0.9 AUC scores.

It is evident from the data presented here that T cell assessment on a population level generates insights about an infection during certain conditions that would, otherwise, not have been feasible with conventional serology. For instance, among the health care workers (the cohort representing high exposure to SARS-CoV-2), groups having negative PCR and serological tests were not distinguishable by their SARS-CoV-2 RBD, NP, and S1-S2 antigen–specific IgG reactivity but showed substantial differences in their T cell metacluster percentages. Health care workers having tested positive by PCR could not be distinguished from their PCR-negative IgG-positive counterparts on the basis of SARS-CoV-2–specific IgG data only, but our celluloepidemiology approach could differentiate these groups. Celluloepidemiology showed also to be uniquely capable to identify “unexposed controls” to actually be “asymptomatic” patients.

In our TCR repertoire analysis, we found that SARS-CoV-2 unique and coronavirus-specific TCRs were present in patients who recovered from COVID-19 more than controls. This highlights that a TCR module in the celluloepidemiology paradigm is useful for differentiating between different infections and backward tracing of pathogen-infected patients thanks to the added layer of TCR specificity, which cannot always be obtained from T cell phenotypes alone. Within the population, there remains residual T cell immune response to other coronaviruses, which bear moderate amino acid conservation with SARS-CoV-2 (*37*). Thus, epitopes recognized by T cells are likely shared between these viruses, leading to this cross-reactivity which has been observed in some studies (*17*, *38*, *39*).

Despite the noteworthy findings, our study has several limitations. One of them is that pre-COVID donors were possibly not the best controls to compare with other cohorts due to the different PBMC isolation methods (i.e., Ficoll was used for pre-COVID PBMC, whereas SepMate tubes and Lymphoprep were used for PBMCs of the other cohorts with distinct centrifugation time and speed in the two protocols). This might have an impact on the composition, quantity, and potentially functional activities of the isolated cells (*40*). In addition, the percentage of individuals with long Covid was notably high compared to other published cohorts (*41*–*43*), which might be due to a selection bias that might have occurred in this retrospective recruitment design. Recruitment via general practitioners and hospitals likely excludes patients with no symptoms (although these will likely be among the household members) and patients with long Covid were likely more motivated to participate. Nevertheless, such selection bias has no impact on the feasibility of the paper or its objectives. Another point that is worth discussing is the feasibility and scalability of celluloepidemiology because such intricate T cell information requires substantial (even labor-intensive) data collection and analysis. This is one of the reasons why, despite all the important insights and benefits that celluloepidemiology can offer, seroepidemiology likely remains more feasible for many studies.

In conclusion, we believe that the proposed celluloepidemiology paradigm is complementary to conventional seroepidemiology in offering high dimensionality, sensitivity, and deep insight into the heterogeneity of human immune response against pathogens. Although celluloepidemiology was introduced and evaluated using the SARS-CoV-2 model in this study, the same concept is highly translatable to different pathogens and infectious diseases in the future.

## METHODS

### Participant recruitment

A total of 582 participants between 18 and 85 years of age were recruited in Belgium from August 2020 until April 2021 via general practitioners, hospitals, and social media. The dominant SARS-CoV-2 strain (in Belgium) was the original Wuhan strain. None of the participants was vaccinated against SARS-CoV-2. Participants in this study were divided into five categories: (i) patients who recovered from COVID-19 (*n* = 168), (ii) household members of the recovered patients (*n* = 27), (iii) controls (*n* = 259), (iv) general practitioners (*n* = 37), and (v) hospital health care workers (*n* = 91). Clinical/demographic details of all participants can be found in table S5. This study was approved by the Antwerp University Hospital IRB (reference number 20/02/003).

Patients who recovered from COVID-19 (referred to as patients in this study) were participants who had recovered from COVID-19 more than 3 months before enrollment and were screened before blood draws to make sure that they were symptom free and in a recovered phase. On their visits, patients were asked to provide proof of positive testing for SARS-CoV-2, either via PCR or serological IgG testing and fever of 38°C or higher without other proven explanations, more than 3 months before recruitment. Patient classification criteria and disease severity (i.e., mild, moderate, and severe infection) were consistent with the COVID-19 case definitions from the US National Health Institute (*44*), World Health Organization (*45*, *46*), and European Centre for Disease Prevention and Control (*47*). Mild cases reported various COVID-19–related manifestations (e.g., fatigue, fever, cough with or without sputum production, anorexia, malaise, myalgia, sore throat, dyspnea, nasal congestion, and headache; rarely diarrhea, nausea, and vomiting) that did not require hospitalization. Moderate cases showed evidence of lower respiratory tract disease, required outpatient hospital visits, and had oxygen saturation ≥ 94% on room air at sea level. Severe cases required hospitalization and had oxygen saturation < 94% on room air at sea level, a ratio of arterial partial pressure of oxygen to fraction of inspired oxygen < 300 mmHg, respiratory frequency > 30 breaths per minute, or lung infiltrates > 50%. Within the patient cohort, there were also “long Covid” cases who reported suffering from the post-acute sequelae of COVID-19 for more than 3 months after recovery (*27*, *28*). Household members were participants who lived in the same household with proven SARS-CoV-2 patients (who tested positive for SARS-CoV-2 by either PCR or IgG and had fever more than 3 months before). Household members did not have a confirmed SARS-CoV-2 infection, either by PCR or IgG.

General practitioners and hospital health care workers (general practitioners and hospital personnel having worked for minimal 4 weeks on COVID-19 wards more than 3 months before sampling) were recruited into five subgroups according to their PCR and IgG test results as well as their symptoms:

1) Group 1: PCR negative or not tested, IgG negative, without fever (no indication of SARS-CoV-2 infection since having contact with patients with COVID-19 more than 3 months before blood donation)

2) Group 2: PCR negative or not tested, IgG negative, with fever

3) Group 3: PCR negative or not tested, IgG positive, without fever

4) Group 4: PCR negative or not tested, IgG positive, with fever

5) Group 5: PCR positive (certain SARS-CoV-2 infection regardless of IgG test results)

Controls were participants who were considered healthy and had no known history of any systemic diseases, including, but not limited to, autoimmune disease, diabetes, kidney or liver disease, congestive heart failure, malignancy, coagulopathy, hepatitis B or C, or HIV. These participants did not have any known household exposure to SARS-CoV-2 nor positive testing (PCR/IgG). Also, included in this study were 24 pre-COVID donors, whose blood was collected for our previous studies between 2015 and 2018, which would be used as unexposed controls, given that SARS-CoV-2 emerged in late 2019.

### Blood collection, processing, and storage

Whole blood from all participants was collected in lithium-heparin and serum separator Vacutainer tubes (BD, catalog no. 367526, 366444) and processed within 6 hours. All blood samples were pseudonymized. To isolate the PBMCs, whole blood in lithium-heparin tubes was diluted 1:2 with phosphate-buffered saline (PBS) (Thermo Fisher Scientific, catalog no. 14190250) and layered into SepMate-50 tubes (STEMCELL Technologies Inc., catalog no. 85460) preloaded with Lymphoprep (STEMCELL Technologies Inc., catalog no. 07861). The SepMate tubes with layered blood and Lymphoprep were spun for 10 min at 1200*g* at room temperature, and the PBMCs were then harvested by collecting the supernatant and washing with PBS. The harvested PBMCs were aliquoted into cryovials in fetal bovine serum (FBS) (Thermo Fisher Scientific, catalog no. 10270-106) with 10% dimethyl sulfoxide (Merck Life Science, catalog no. D2650) and stored in liquid nitrogen until use. PBMCs from pre-COVID controls were processed like described previously (*48*).

Blood in serum separator tubes was allowed to clot for 30 to 60 min and then centrifuged for 10 min at 1200*g* at room temperature. After centrifugation, serum located above the polymer barrier was transferred to cryovials and stored at −80°C for Luminex assays.

### Marker selection with mass cytometry

Mass cytometry, or CyTOF, was performed using an extensive panel of isotope-tagged antibodies for T cells to select markers that could display the most distinction in T cell activation between controls and patients for the next flow cytometry measurements. Only PBMCs from 45 donors were used in the first CyTOF runs including 30 patients and 15 pre-COVID donors (table S6).

Cryopreserved PBMCs were thawed at 37°C in a water bath and then diluted in pre-warmed AIM-V medium (Thermo Fisher Scientific, catalog no. 12055091) with 5% FBS and subsequently centrifuged for 5 min at 300*g* at room temperature. After the supernatant was discarded, ~1 to 2 million thawed PBMCs were used for each stimulating condition: (i) stimulated with SARS-CoV-2 membrane protein (Prot_M) PepTivator (Miltenyi Biotec, catalog no. 130-126-702) at 2 μg/ml, (ii) stimulated with MHC class I–specific pool, and (iii) MHC class II–specific pool (JPT Peptide Technologies, custom synthesized) also at 2 μg/ml. Unstimulated samples were used as negative controls. Samples stimulated with phytohaemagglutinin (PHA) at 5 μg/ml were used as positive controls. The custom-synthesized peptides were based on a curated list of known SARS-CoV-2 epitopes, extracted from various existing studies (*49*–*53*) using the Enzyme-Linked Immunospot assay (ELISpot), in silico prioritization and TCR-epitope simulation. Those epitopes that were most consistently reported as immunogenic across different studies were retained. In addition, these epitopes were compared to a list of 119 Nidovirales genomes from the Corona OMA Orthology Database (*54*) to identify which were SARS-CoV-2 specific. A list of these peptides and their amino acid sequences is displayed in table S7. CD40 (Miltenyi Biotec, catalog no. 130-094-133) and CD28 (BD, catalog no. 555725) were both added at 1 μg/ml to each culture, and PBMCs in all four stimulating conditions were incubated for 16 hours at 37°C. After incubation, staining started with CD45 barcodes (i.e., CD45 antibodies conjugated with ^106^Cd, ^110^Cd, ^111^Cd, ^113^Cd, ^114^Cd, and ^116^Cd) for 30 min, then isotope-tagged antibodies (table S1) for another 30 min, and ^103^Rh-intercalator (Standard BioTools Inc., catalog no. 201103A) for 15 min. Cells were subsequently fixed for 10 min in 4% paraformaldehyde (Thermo Fisher Scientific, catalog no. 28908) before being stained with Ir-intercalator (Standard BioTools Inc., catalog no. 201192A) overnight at 4°C. Measurements were performed on the Helios instrument (Standard BioTools Inc.) at the KU Leuven Flow and Mass Cytometry Facility.

CyTOF output data were normalized and debarcoded in the CyTOF software (Standard BioTools Inc.). Then, FlowJo (FlowJo LLC.) was used for data gating (gating strategies are displayed in fig. S10). Total numbers of CD4^+^ and CD8^+^ T cells as well as numbers of T cells with high expression of activation markers (i.e., CD38, OX40, TIGIT, PD-1, CTLA-4, CD69, Fas, CD154, TIM-3, CD57, HLA-DR, LAG-3, CD127, and CD137) in stimulated samples relative to T cells’ expression in unstimulated controls were then exported from FlowJo, and percentages of antigen-specific T cells were analyzed in R. Boruta package (*25*), a feature selection algorithm, was used to find the optimal markers that could distinguish patients with COVID-19 from pre-COVID participants and differentiate patient subgroups (i.e., mild, moderate, and severe).

### Flow cytometry measurements

In the first set of experiments, PBMCs from controls, patients, household members, hospital health care workers, general practitioners, and pre-COVID donors were used for flow cytometry measurements. Cells were thawed and stimulated as previously described for CyTOF. After stimulation, cells were stained for 30 min with a panel of 17 fluorescent antibodies (table S2) in Brilliant Stain Buffer (BD, catalog no. 563794) before being analyzed on the NovoCyte Quanteon flow cytometer (Agilent Technologies Inc.). In the second set of experiments, only PBMCs from controls, patients, and household members were analyzed. Cells were stimulated with a combination of MHC class I– and class II–specific pool (JPT Peptide Technologies, custom synthesized) at 3 μg/ml. Unstimulated samples were used as negative controls. Samples stimulated with PHA at 5 μg/ml were used as positive controls. After stimulation, cells were prepared following the overnight staining protocol (*55*). Briefly, cells were first stained with the fixable viability stain for 15 min. Then, they were fixed for 10 min in 0.4% paraformaldehyde (BioLegend, catalog no. 420801) at room temperature. Afterward, they were stained with a panel of 12 fluorescent antibodies (table S3) overnight at 4°C. Measurements were also performed on the NovoCyte Quanteon flow cytometer.

Data compensation, transformation, and automated gating were performed using R packages flowCore, flowWorkspace, and openCyto. FlowSOM was performed following the authors’ instructions (*35*) to cluster cells for immunophenotyping. Like previous CyTOF data analysis, total numbers of CD4^+^ and CD8^+^ T cells as well as numbers of T cells with high expression of functional markers (i.e., CD38, CD69, Fas, OX40, CD137, CD154, LAG-3, and TIGIT) in stimulated samples relative to T cells’ expression in unstimulated controls were exported (figs. S11 and S12). The percentages of antigen-specific T cells were used to develop classification models. The data were split into training data and held-out testing data. Random forest classifiers (*36*) were trained on the basis of the training data using the R package randomForest. Leave-one-out cross-validation was used to evaluate classifiers’ performance. Receiver operating characteristic (ROC) curves and the AUC were used using the R package pROC (*56*) to evaluate the classifier performance. AUC values were displayed as means ± confidence interval (95%).

### Luminex assay

Luminex assays were performed on serum isolated from blood in BD Vacutainer serum separator tubes following the same procedure as in a previous publication (*6*). The lyophilized recombinant NP, RBD, and spike (S1-S2) antigens (Sino Biological Inc.) were resuspended in a buffer according to the manufacturer’s instructions and stored until use. One microgram of RBD and S1-S2 and 2 μg of NP were coupled to 1.25 × 10^6^ paramagnetic MAGPLEX COOH-microsphere beads (Luminex Corporation) for IgG detection. Bead working solution was prepared in a mixture of PBS and bovine serum albumin (BSA) with a concentration of 2000 beads per antigen per well. Serum samples were diluted 1:300 and added to each well together with the bead working solution in a final volume of 150 μl. Plates were incubated at room temperature for 2 hours in the dark and then washed with 200 μl per well of PBS-BSA buffer. After incubation with biotin-labeled anti-human IgG secondary antibody and streptavidin-R-phycoerythrin conjugate, reactions were read on a Luminex 100/200 analyzer (Luminex Corporation). Results were expressed as median fluorescent intensities and normalized for further statistical analyses. When considering combinations of (RBD-NP and S1S2 antigens), the test has the following sensitivities at the 99% specificity cutoff: severe-recent (95%), recent-mild (99%), and old-mild (96%).

### T cell receptor sequencing

TCR-seq was performed on enriched CD8^+^ T cells. Cryopreserved PBMCs were thawed at 37°C in a water bath, then diluted in pre-warmed AIM-V medium supplemented with 5% FBS, and subsequently centrifuged for 5 min at 300*g* at room temperature. After the supernatant was discarded, cells were washed with precooled MACS Separation Buffer (Miltenyi Biotec, catalog no. 130-091-221) and centrifuged for 5 min at 300*g*, after which the supernatant was discarded. Magnetic cell separation was then performed using MS columns, OctoMACS Separator, and CD8 anti-human MicroBeads (Miltenyi Biotec, catalog nos. 130-042-201, 130-042-108, and 130-045-201, respectively) following the supplier’s instructions. After enrichment, ~200,000 CD8^+^ T cells were used for RNA extraction via the Quick-RNA Microprep Kit (Zymo Research, catalog no. R1050). TCR libraries were prepared from the extracted RNA using the QIAseq Immune Repertoire RNA Library Kit (QIAGEN, catalog no. 333705) following the supplier’s instructions. The amplified TCR libraries were equimolarly pooled and sequenced on NextSeq 500 (Illumina).

### TCR data analysis

TCR clonotype annotation was performed using MiXCR v.3.0.13 with the default input parameters (*57*). Epitope specificity of the TCRs was predicted with TCRex (*58*). SARS-CoV-2 epitopes were considered coronavirus common if they were found in protein sequence data of at least 2 of the 119 *Nidovirales* species as had been previously reported (*59*). Clustering of TCRs with similar CDR3 sequences was performed for CD8^+^ TCR repertoire of every individual separately using ClusTCR v.1.0.2 with default parameters (*60*). Clusters that contained TCRs, for which specificity had been identified with TCRex, were annotated with specificity of those TCRs. Only clusters containing TCRs with the same predicted (or unknown) specificity were retained for further analysis. For the remaining clusters, all TCRs within one cluster were considered to have the same specificity. After clustering, counts of annotated TCRs and clusters were compared between different groups.

### Statistical analyses

The two-sided Fisher’s exact test was performed in R to evaluate the significance of T cells’ response to membrane (M) and MHC class I– and class II–specific peptides compared to unstimulated T cells. Statistical significance analyses and multiple comparison correction were done in R or GraphPad Prism (GraphPad Software Inc.). Two-group comparison was done with Mann-Whitney test. Multiple groups were compared by Kruskal-Wallis test. These symbols indicate *P* values of statistical significance: *P ≤ 0.05; **P ≤ 0.01; ***P ≤ 0.001; ****P ≤ 0.0001.
