# Supplementary material for: Celluloepidemiology—A paradigm for quantifying infectious disease dynamics on a population level
Source: Sci Adv. 2025 May 16;11(20):eadt2926. doi: 10.1126/sciadv.adt2926 (PMC12083542; doi:10.1126/sciadv.adt2926)
Supplement: Supplementary file 1 — Figs. S1 to S13 Tables S1 to S3 Legends for tables S4 to S9 [file sciadv.adt2926_sm.pdf]

Science Advances
AAAS

Supplementary Materials for

Celluloepidemiology—A paradigm for quantifying infectious disease dynamics on a population level

My K. Ha *et al.*

Corresponding author: My K. Ha, my.ha@uantwerp.be; Benson Ogunjimi, benson.ogunjimi@uantwerp.be

Sci. Adv. **11**, eadt2926 (2025)
DOI: 10.1126/sciadv.adt2926

The PDF file includes:

Figs. S1 to S13
Tables S1 to S3
Legends for tables S4 to S9

Other Supplementary Material for this manuscript includes the following:

Tables S4 to S9

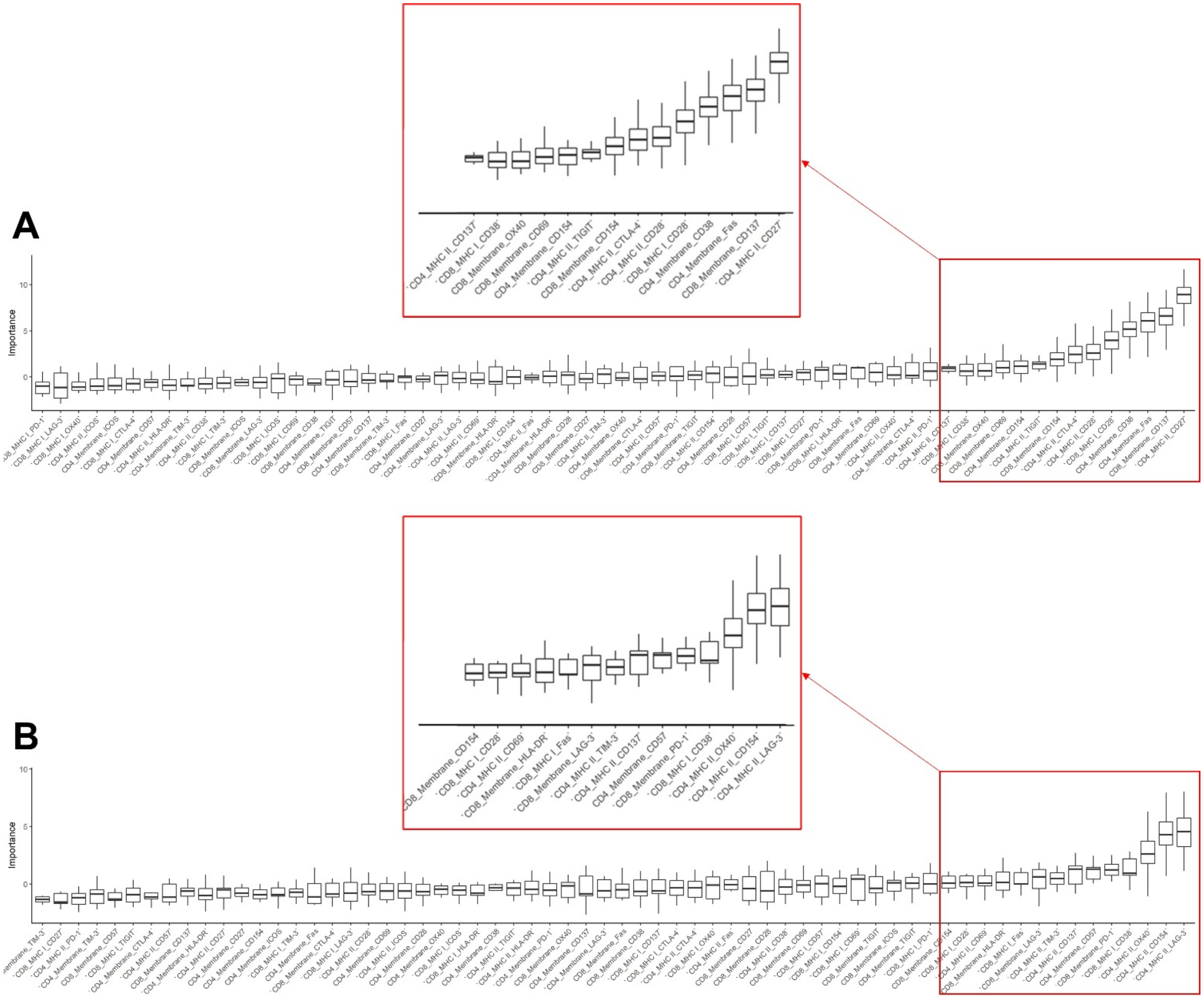

Fig. S1. Feature importance estimated by the Boruta algorithm. (**A**) Comparison between recovered COVID-19 patients and control; (**B**) Comparison between patients with mild, moderate, and severe COVID-19.

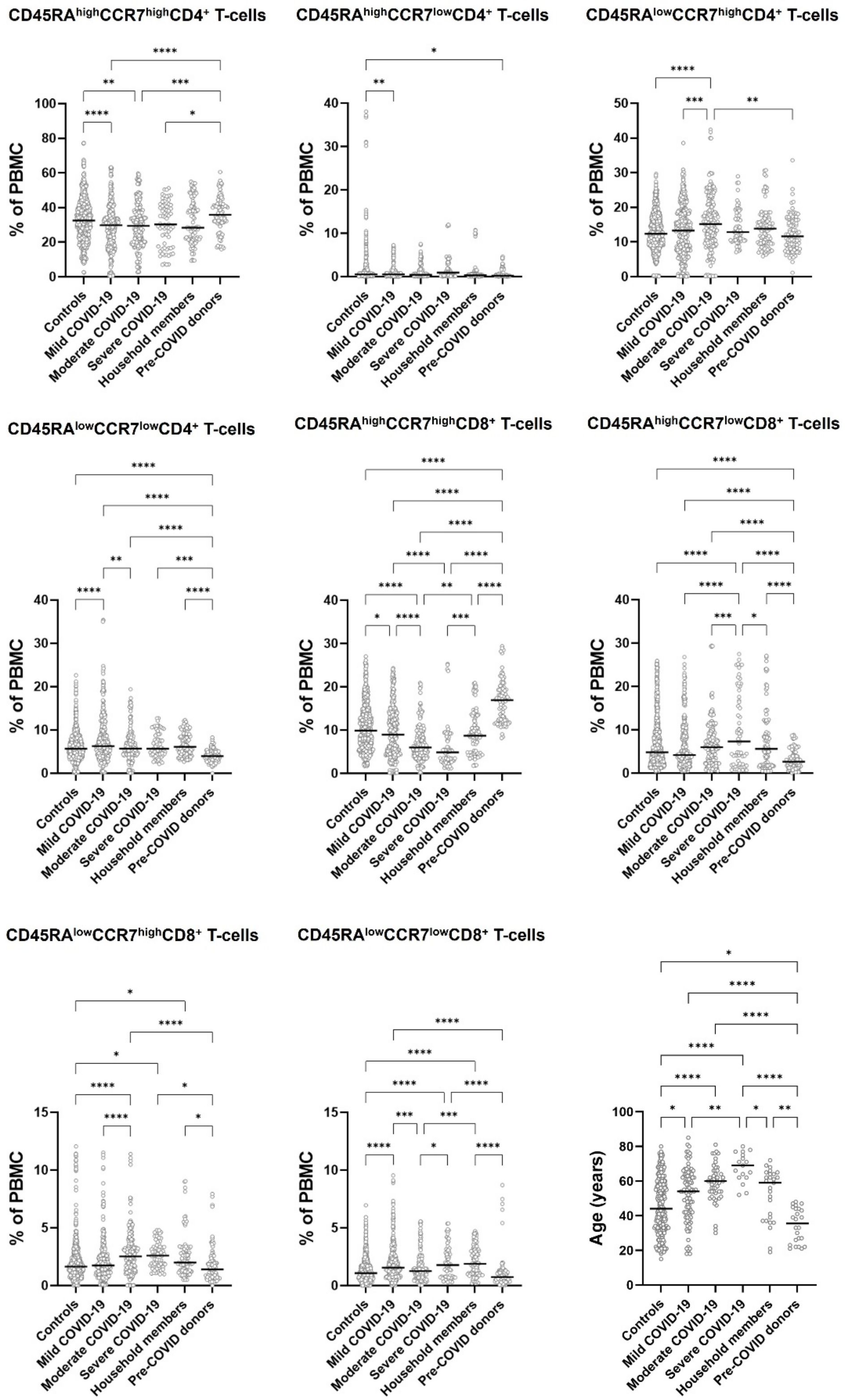

Fig. S2. Comparisons of CD4⁺ and CD8⁺ T-cells' percentages and age between recovered COVID-19 patients, their household members, controls, and pre-COVID donors. Horizontal lines represent median values of each group. Statistical significance was analyzed by Kruskal-Wallis test with Dunn's correction. $^{*}p \leq 0.05$, $^{**}p \leq 0.01$, $^{***}p \leq 0.001$, $^{****}p \leq 0.0001$.

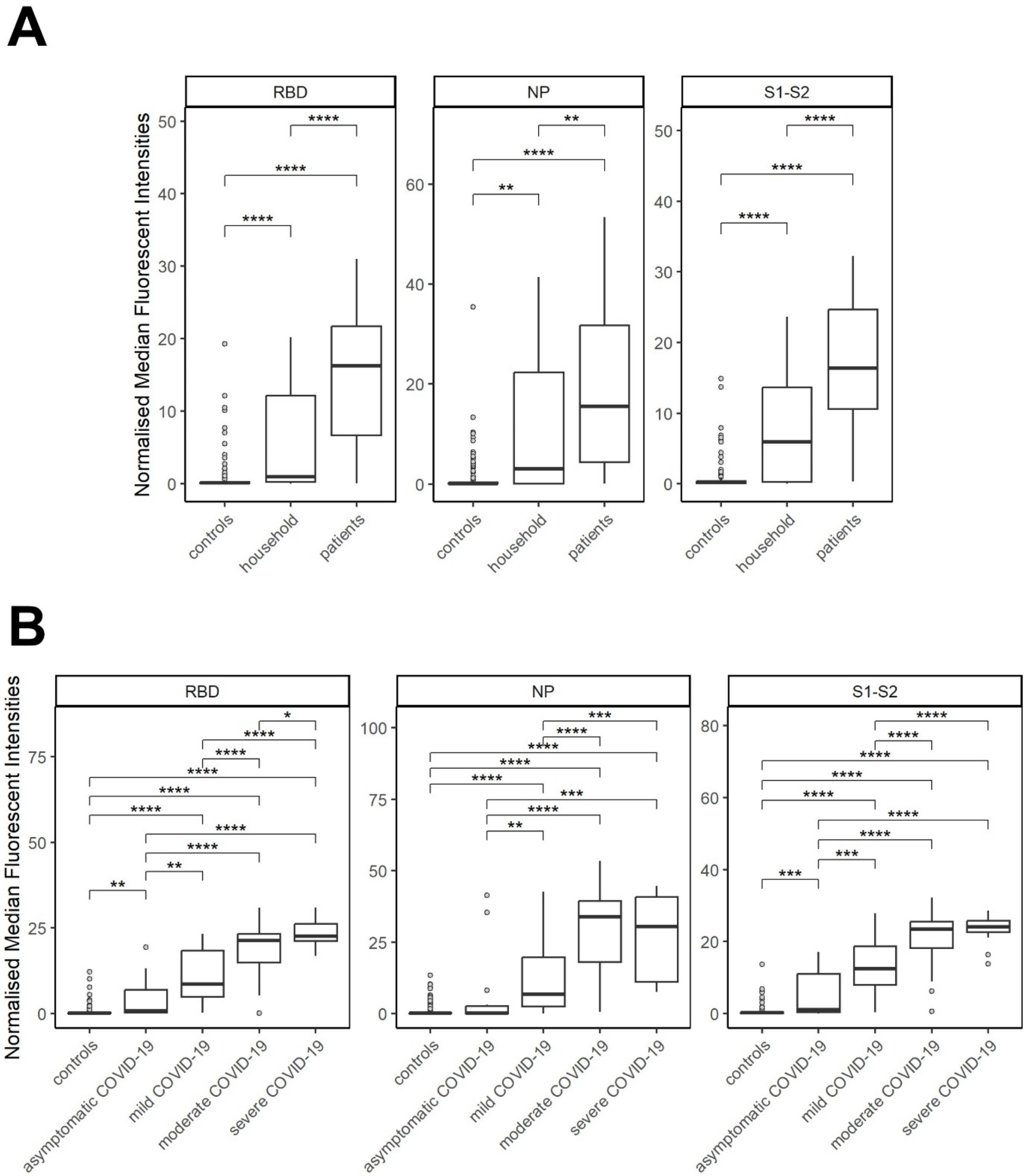

Fig. S3. Levels of SARS-CoV-2 RBD, NP, and S1-S2 antigen specific IgG in controls, patients, and household members. (A) Comparisons between controls and patients; (B) Comparisons between controls, asymptomatic, mild, moderate, and severe patients. Horizontal lines represent median values of each group. Statistical significance was analyzed by Mann-Whitney test or Kruskal-Wallis test with Bonferroni correction. *$p \leq 0.05$, **$p \leq 0.01$, ***$p \leq 0.001$, ****$p \leq 0.0001$.

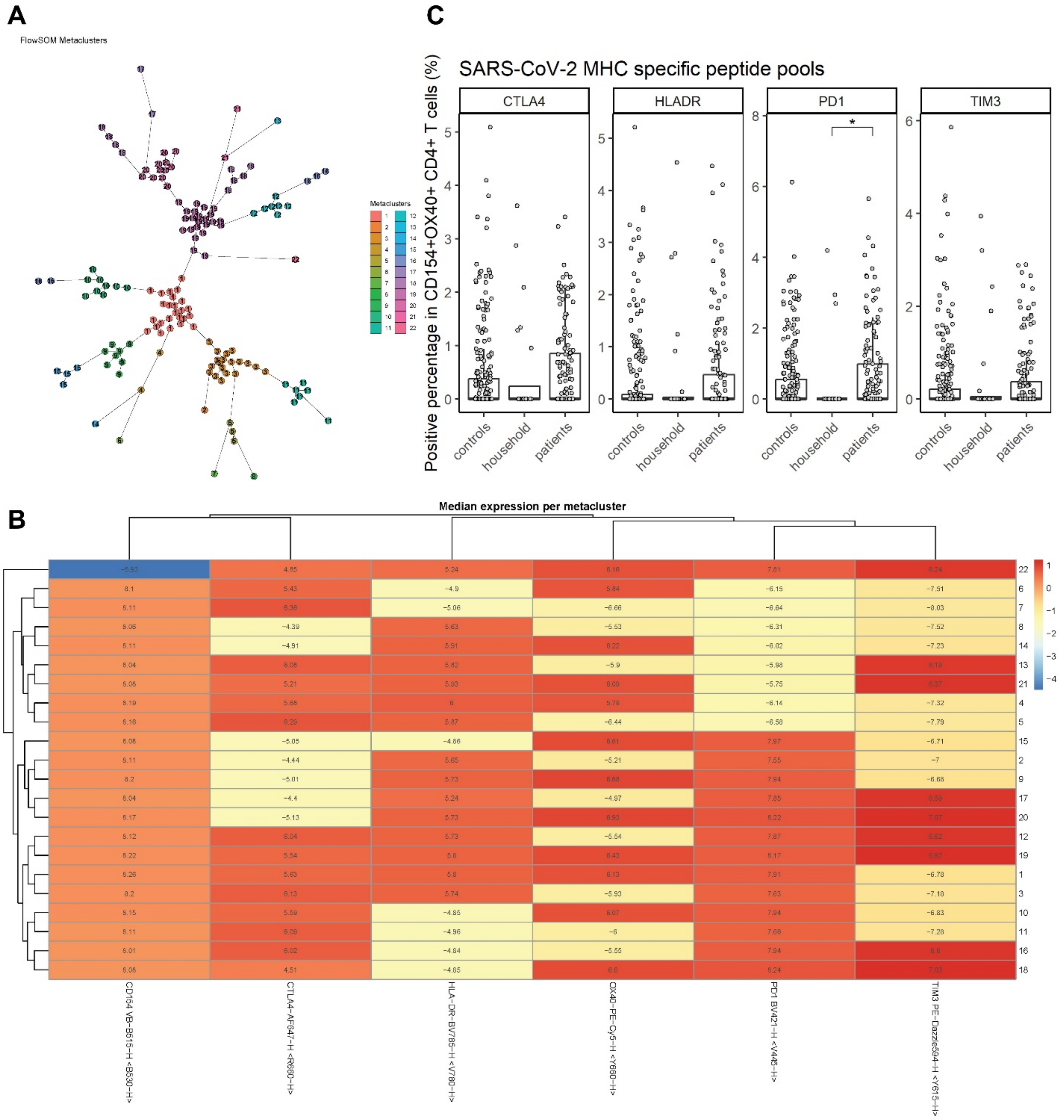

Fig. S4. Further comparison of SARS-CoV-2 specific CD4+ T-cell immune response between controls, patients, and household members. (A) FlowSOM metaclusters. (B) Median expression of different markers in each metacluster. (C) Net percentages of T-cells with positive expression of functional markers out of the total numbers of $CD154^{high}OX40^{high}$ $CD4^{+}$ T-cells post-stimulation (normalized with unstimulated controls). Horizontal lines represent median values of each group. Statistical significance was analyzed by Kruskal-Wallis test with Bonferroni correction. $^{*}p \leq 0.05$, $^{**}p \leq 0.01$, $^{***}p \leq 0.001$, $^{****}p \leq 0.0001$.

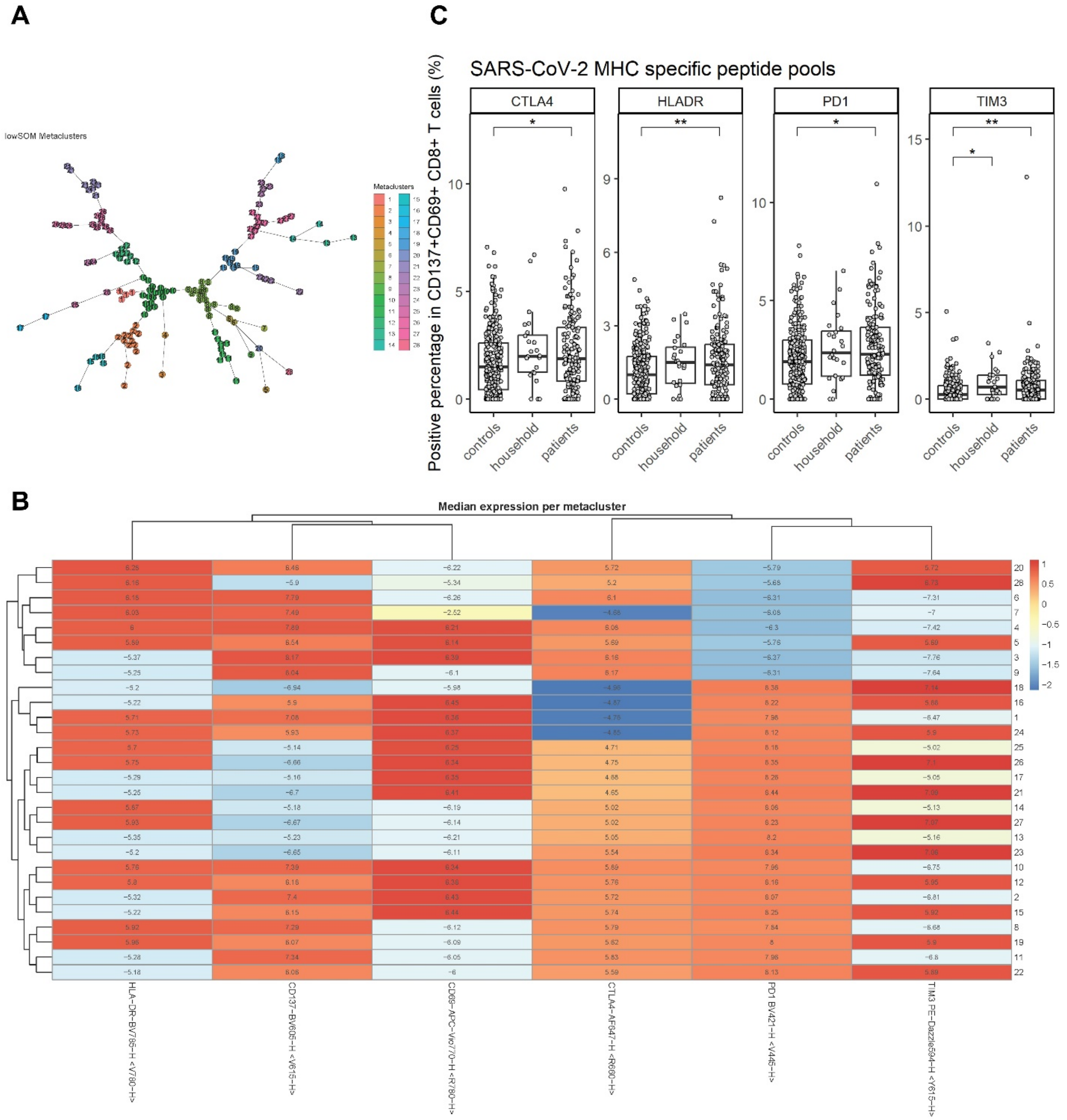

Fig. S5. Further comparison of SARS-CoV-2 specific CD8^{+} T-cell immune response between controls, patients, and household members. (A) FlowSOM metaclusters. (B) Median expression of different markers in each metacluster. (C) Net percentages of T-cells with positive expression of functional markers out of the total numbers of CD137^{high}CD69^{high} CD8^{+} T-cells post-stimulation (normalized with unstimulated controls). Horizontal lines represent median values of each group. Statistical significance was analyzed by Kruskal-Wallis test with Bonferroni correction. *p $\leq$ 0.05, **p $\leq$ 0.01, ***p $\leq$ 0.001, ****p $\leq$ 0.0001.

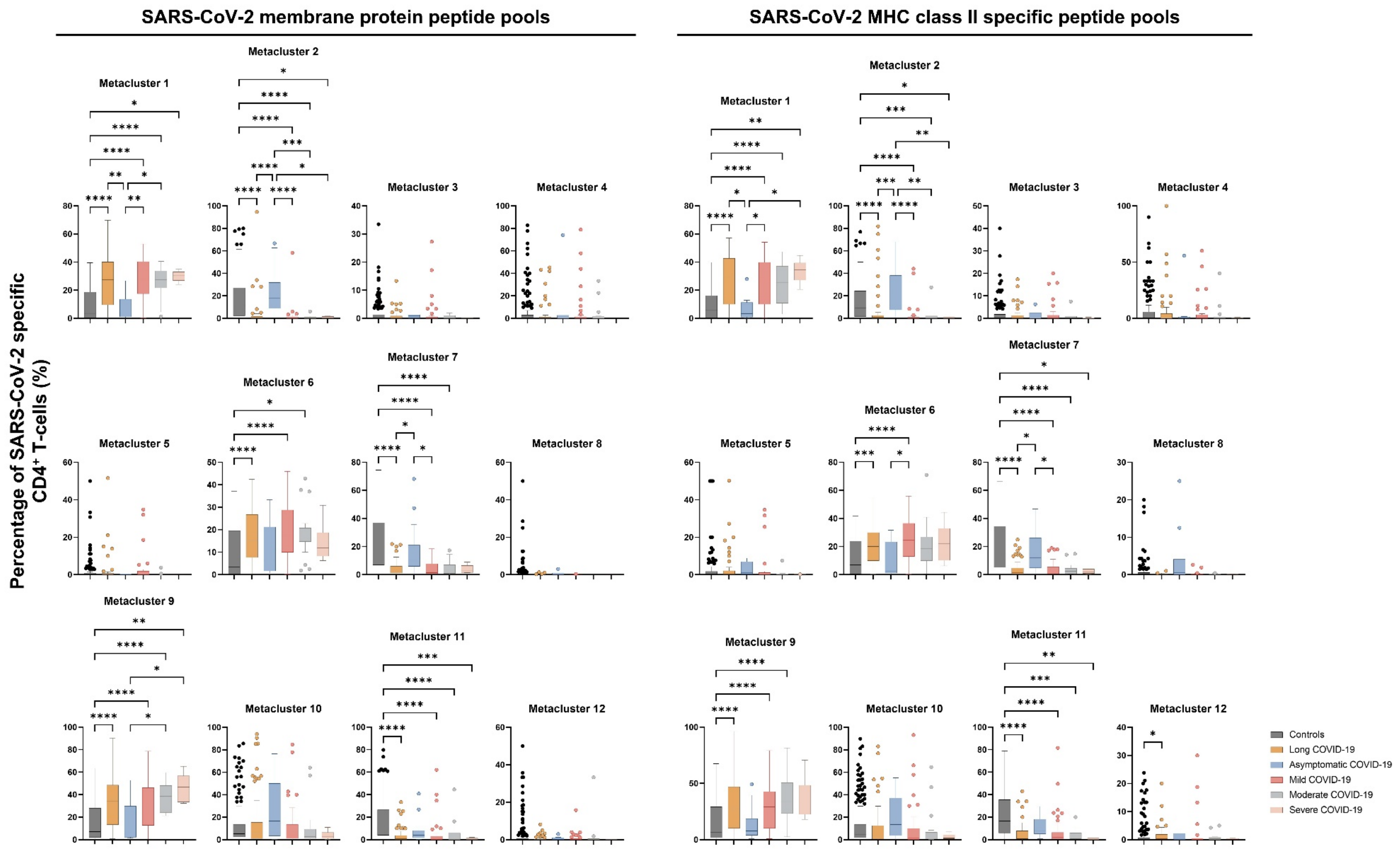

Fig. S6. Percentage of SARS-CoV-2 specific CD4⁺ T-cells of long COVID patients compared to controls and other patient groups in each metacluster of the FlowSOM model in Fig. 3. Horizontal lines represent median values of each group. Statistical significance was analyzed by Kruskal-Wallis test with Dunn's correction. $^{*}p \leq 0.05$, $^{**}p \leq 0.01$, $^{***}p \leq 0.001$, $^{****}p \leq 0.0001$.

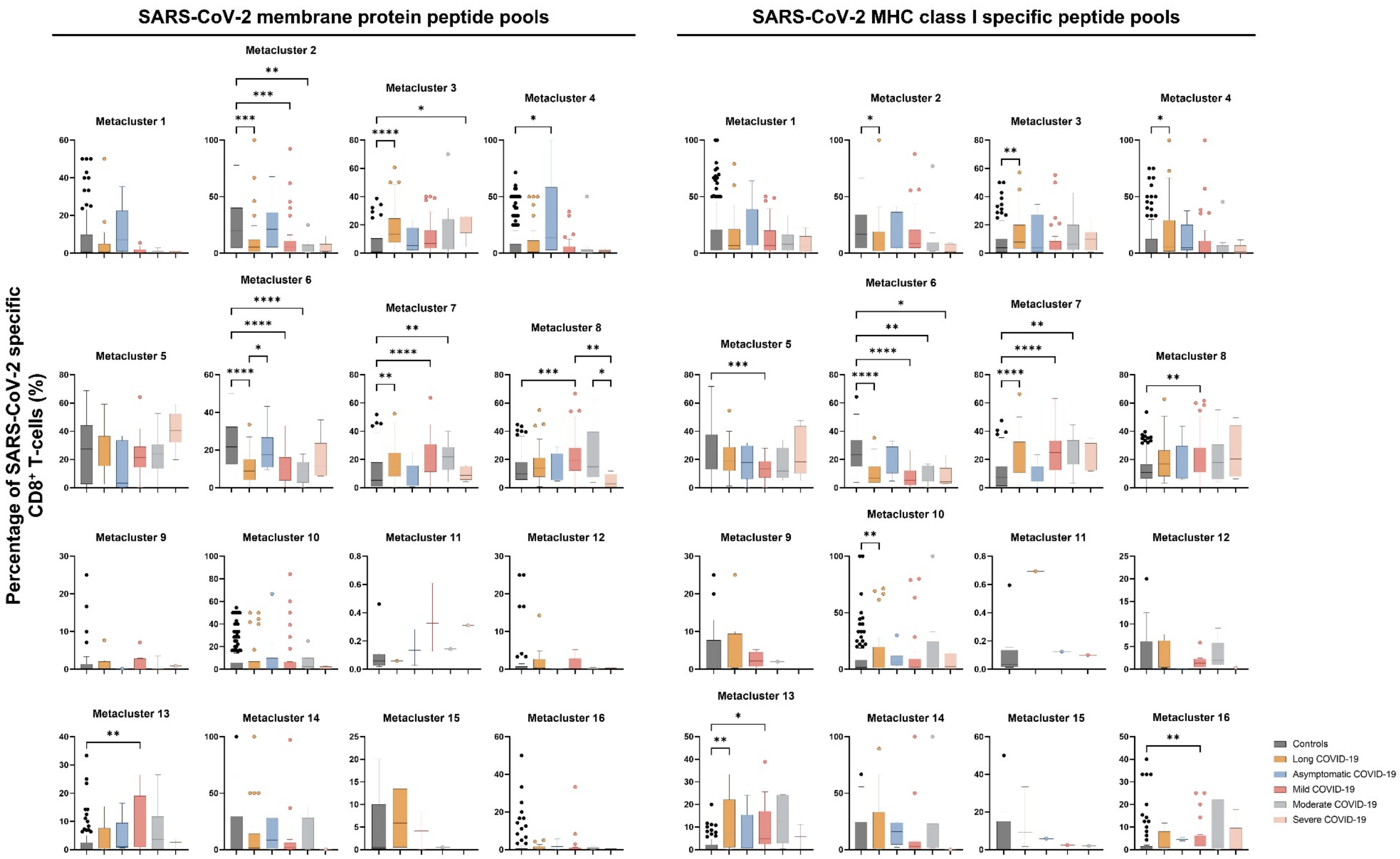

Fig. S7. Percentage of SARS-CoV-2 specific CD8+ T-cells of long COVID patients compared to controls and other patient groups in each metacluster of the FlowSOM model in Fig. 4. Horizontal lines represent median values of each group. Statistical significance was analyzed by Kruskal-Wallis test with Dunn's correction. $^*p \leq 0.05$, $^{**}p \leq 0.01$, $^{***}p \leq 0.001$, $^{****}p \leq 0.0001$.

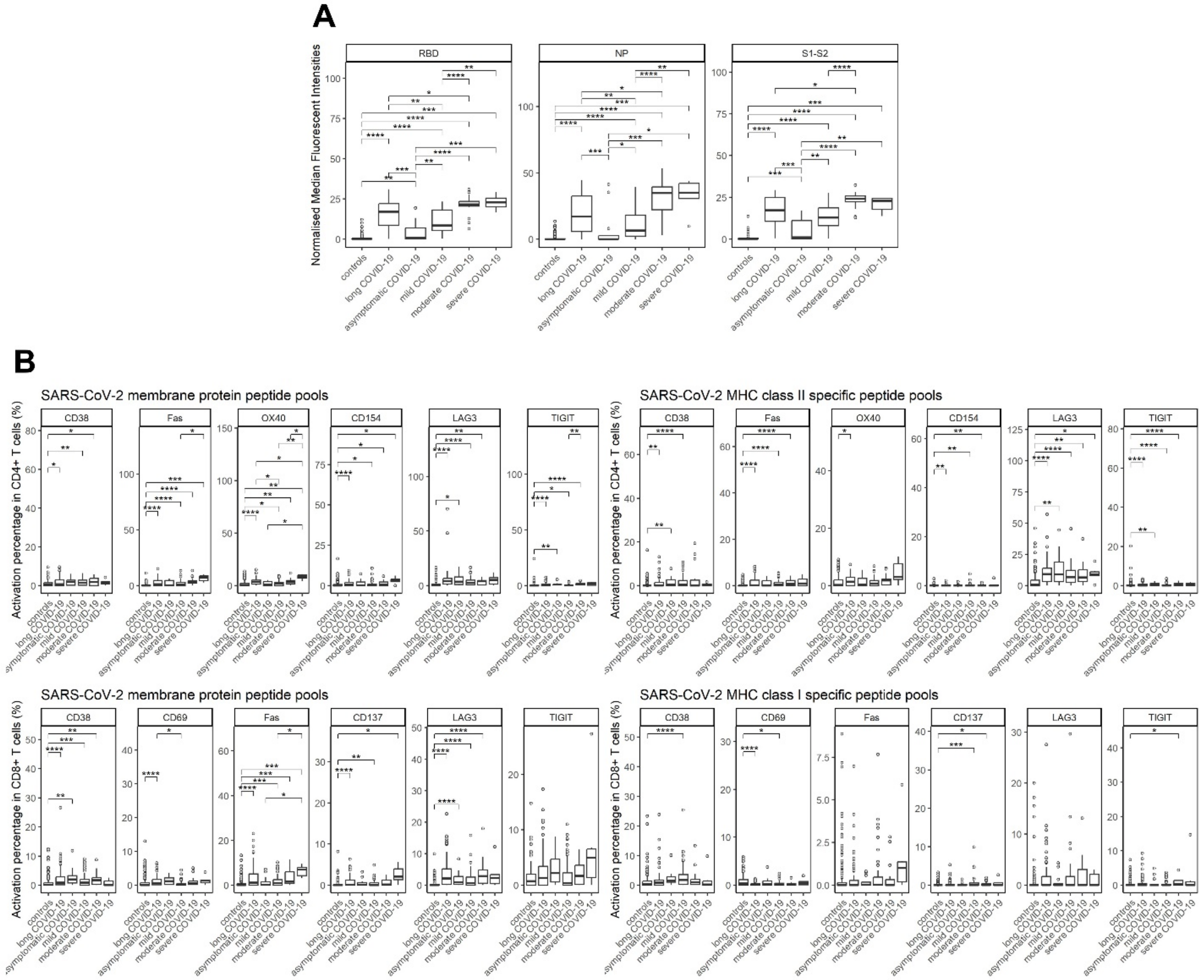

Fig. S8. Humoral and cellular immune responses of long COVID patients compared to controls and other patient groups. (A) Levels of SARS-CoV-2 RBD, NP, and S1-S2 antigen specific IgG. (B) Net percentages of T-cells with positive expression of functional markers out of the total numbers of $CD4^+$ and $CD8^+$ T-cells post-stimulation (normalized with unstimulated controls). Horizontal lines represent median values of each group. Statistical significance was analyzed by Kruskal-Wallis test with Bonferroni correction. $*p \leq 0.05$, $**p \leq 0.01$, $***p \leq 0.001$, $****p \leq 0.0001$.

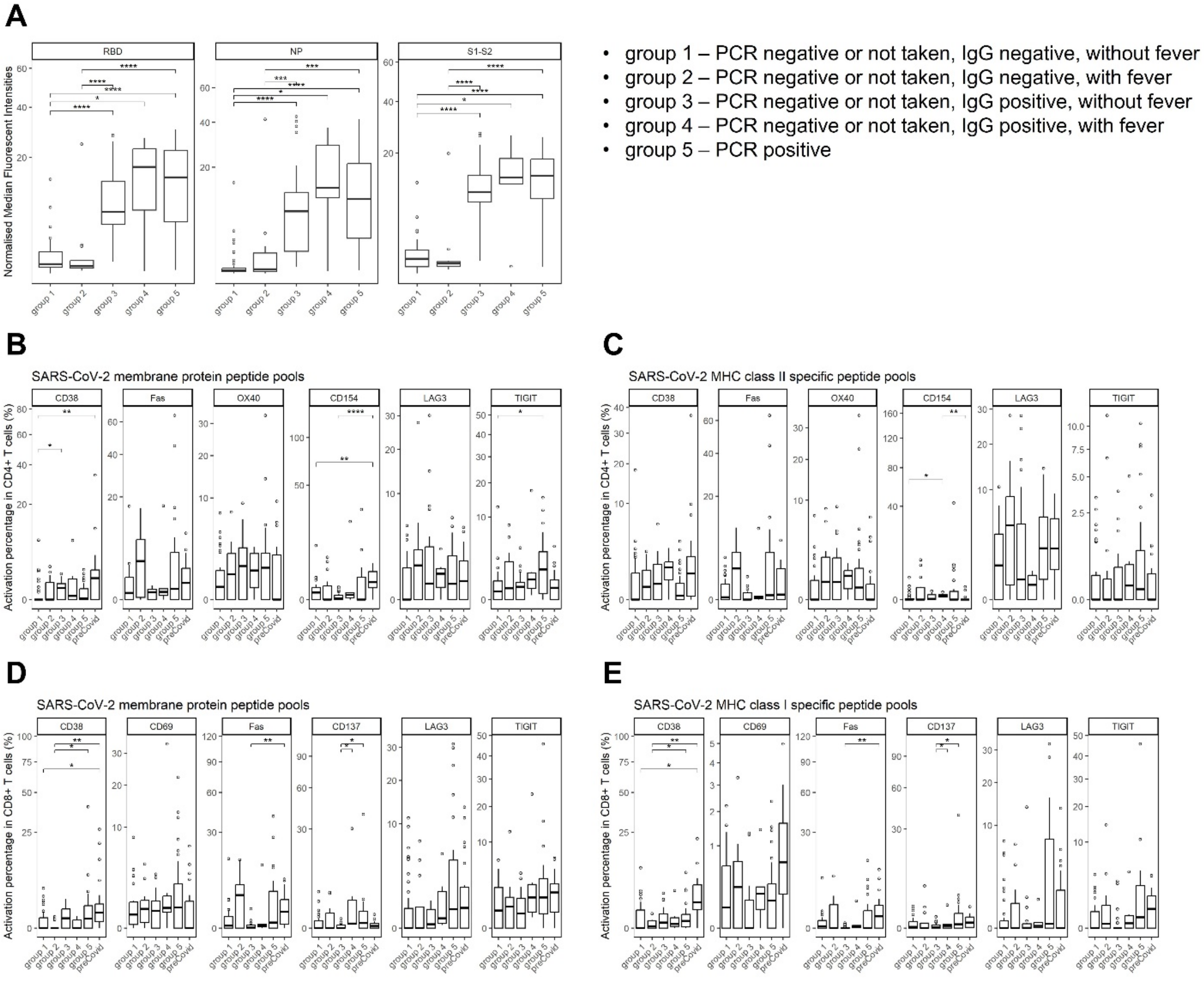

Fig. S9. Humoral and cellular immune responses in healthcare workers against SARS-CoV-2. (A) Levels of SARS-CoV-2 RBD, NP, and S1-S2 antigen specific IgG in healthcare workers. (B–E) Net percentages of T-cells with positive expression of functional markers out of the total numbers of $CD4^+$ and $CD8^+$ T-cells post-stimulation (normalized with unstimulated controls). Y-axes are square-root transformed. Horizontal lines represent median values of each group. Statistical significance was analyzed by Kruskal-Wallis test with Bonferroni correction. $^*p \leq 0.05$, $^{**}p \leq 0.01$, $^{***}p \leq 0.001$, $^{****}p \leq 0.0001$.

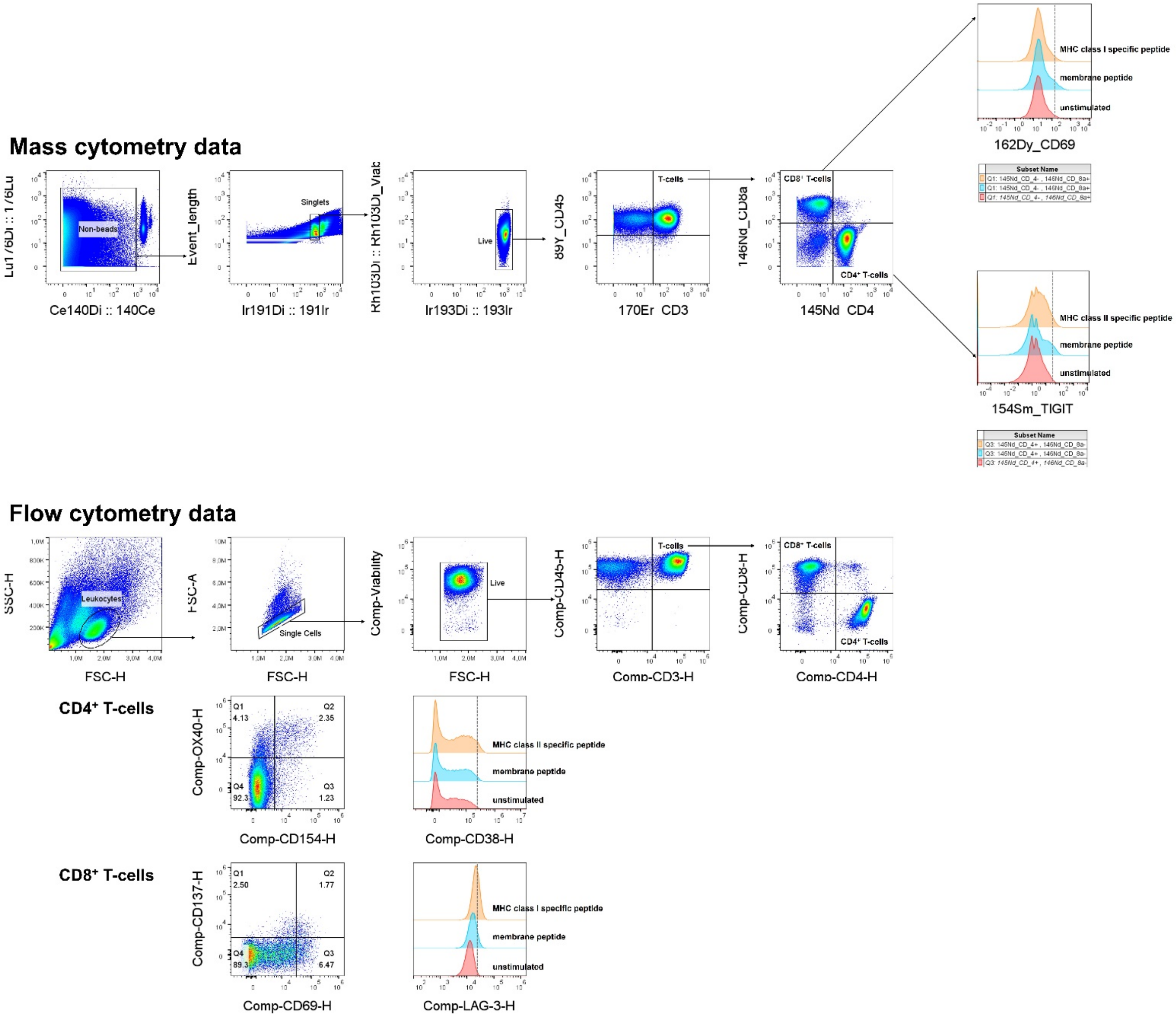

Fig. S10. Example of the applied gating strategies. Upper panel: gating strategy for mass cytometry data based on atomic mass intensity of isotope-tagged antibodies. Lower panel: gating strategy for flow cytometry data based on fluorescence intensity of fluorochrome-tagged antibodies.

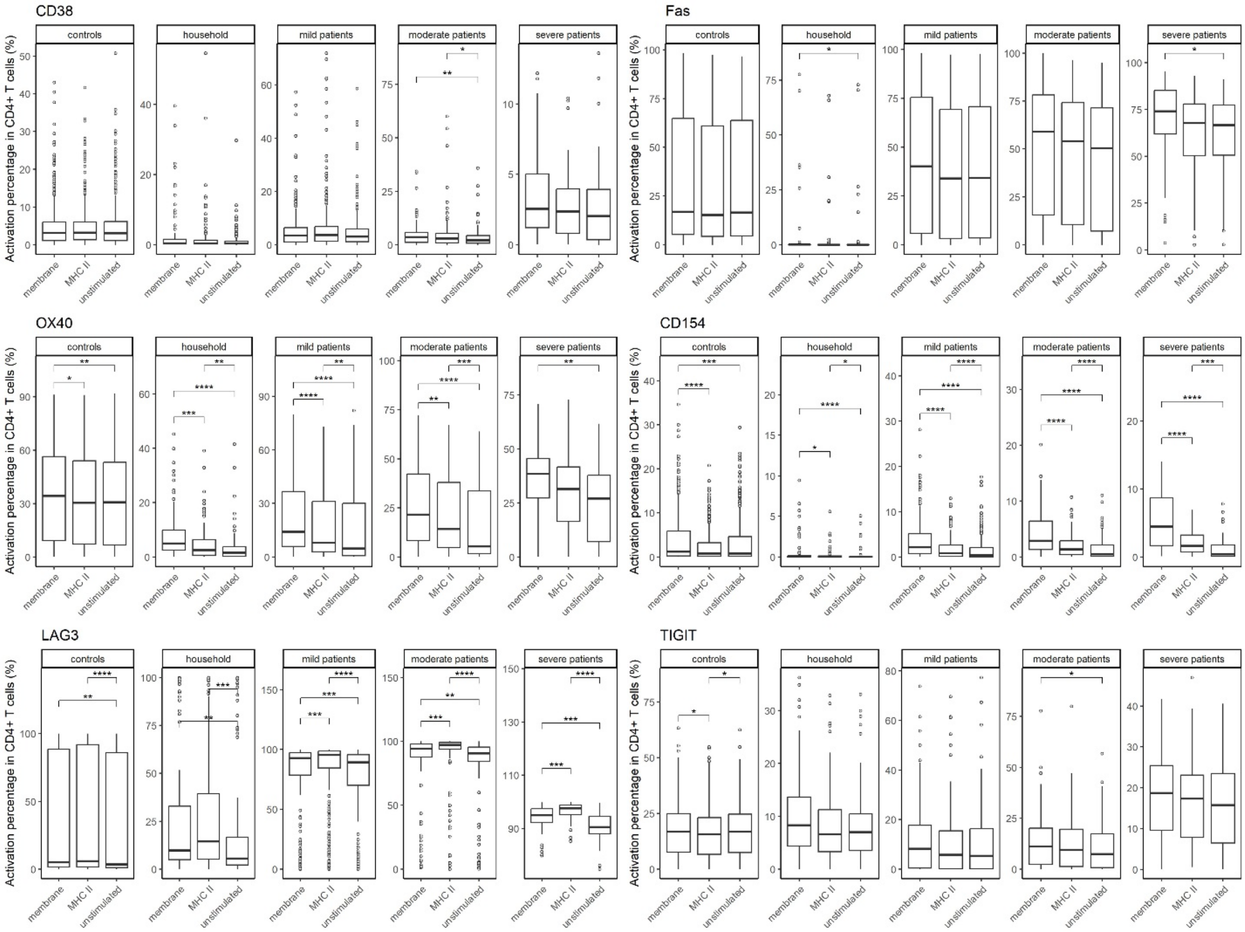

Fig. S11. Percentages of CD4^{+} T-cells with positive expression of functional markers after incubation with different stimuli. Horizontal lines represent median values of each group. Statistical significance was analyzed by Kruskal-Wallis test with Bonferroni correction. $*p \leq 0.05$, $**p \leq 0.01$, $***p \leq 0.001$, $****p \leq 0.0001$.

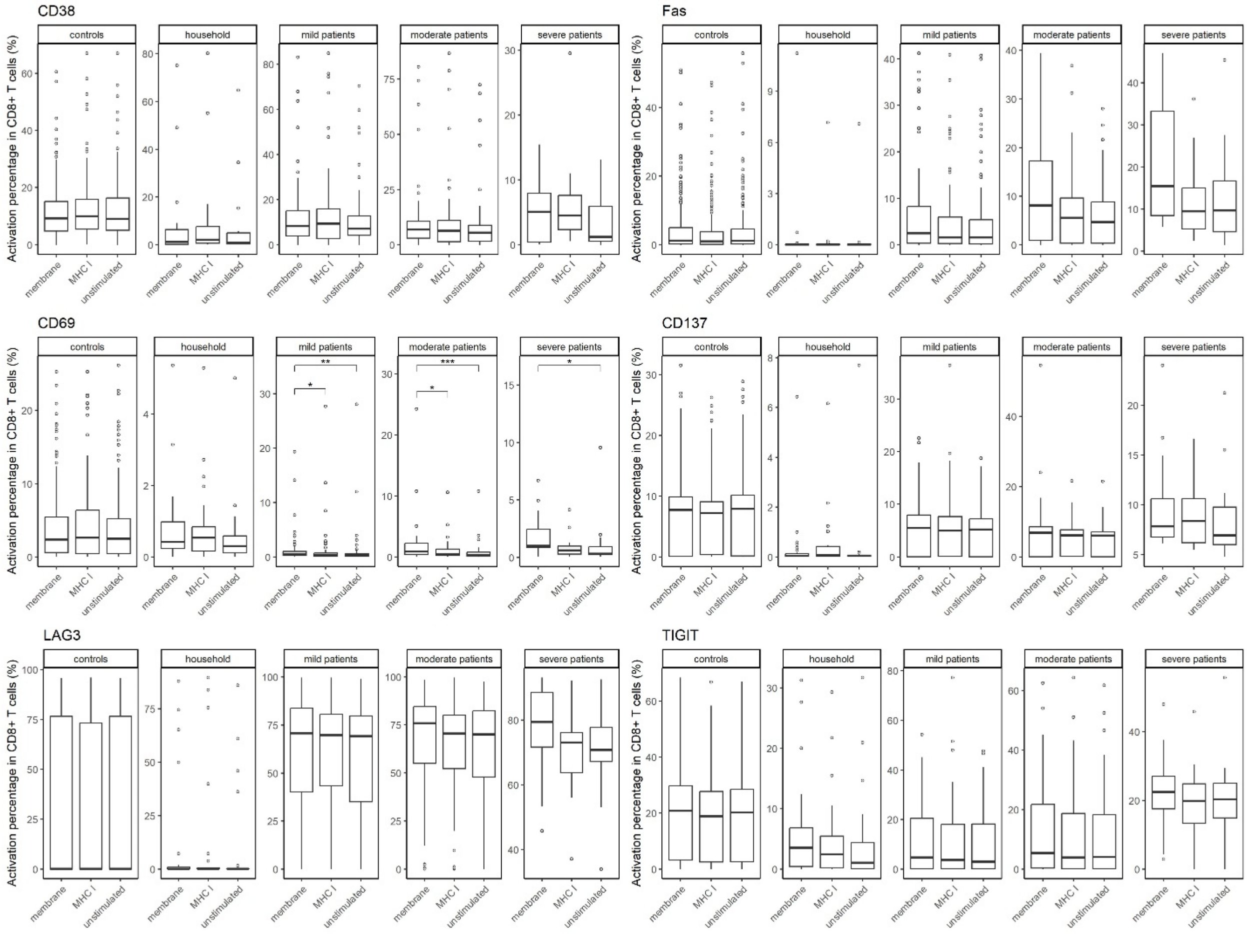

Fig. S12. Percentages of CD8⁺ T-cells with positive expression of functional markers after incubation with different stimuli. Horizontal lines represent median values of each group. Statistical significance was analyzed by Kruskal-Wallis test with Bonferroni correction. *$p \leq 0.05$, **$p \leq 0.01$, ***$p \leq 0.001$, ****$p \leq 0.0001$.

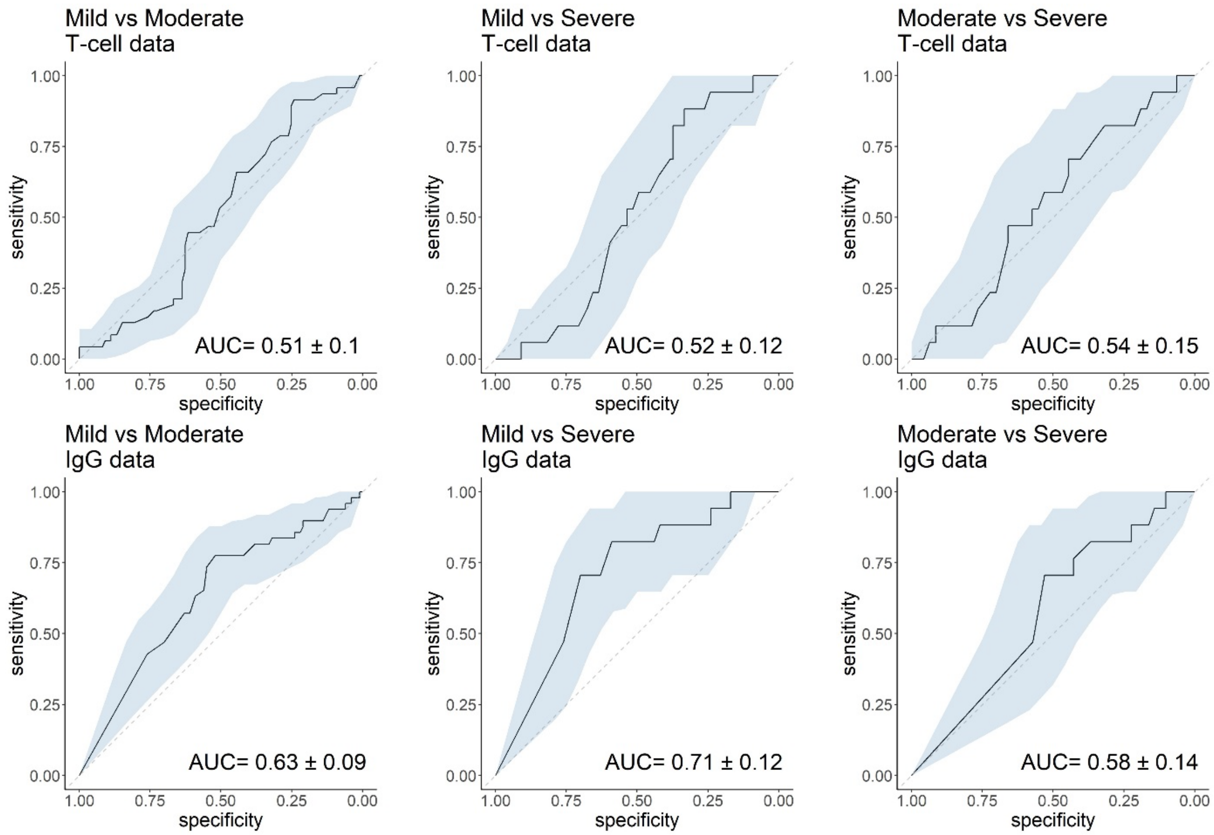

Fig. S13. ROCs and AUCs from leave-one-out cross-validation. Classifiers were developed using Random Forest algorithm based on T-cell data and IgG data.

Table S1. CyTOF antibody panel. All antibodies in this panel were supplied by Standard BioTools Inc.

Isotope	Target	Clone	Catalogue Number
^{106}Cd	CD45	HI30	3106001
^{110}Cd	CD45	HI30	3110001
^{111}Cd	CD45	HI30	3111001
^{113}Cd	CD45	HI30	3113001
^{114}Cd	CD45	HI30	3114001
^{116}Cd	CD45	HI30	3116001
^{89}Y	CD45	HI30	3089003
^{141}Pr	CD196/CCR6	G034E3	3141003
^{142}Nd	CD19	HIB19	3142001
^{143}Nd	CD278/ICOS	C398.4A	3143025
^{144}Nd	CD38	HIT2	3144014
^{145}Nd	CD4	RPA-T4	3145001
^{146}Nd	CD8a	RPA-T8	3146001
^{147}Sm	CD11c	Bu15	3147008
^{149}Sm	CD25/IL-2R	2A3	3149010
^{150}Nd	CD134/OX40	ACT35	3150023
^{151}Eu	CD14	M5E2	3151009
^{153}Eu	CD45RA	HI100	3153001
^{154}Sm	TIGIT	MBSA43	3154016
^{155}Gd	CD279/PD-1	EH12.2H7	3155009
^{156}Gd	CD183/CXCR3	G025H7	3156004
^{158}Gd	CD194/CCR4	L291H4	3158032
^{159}Tb	CD197/CCR7	G043H7	3159003
^{160}Gd	CD28	CD28.2	3160003
^{161}Dy	CD152/CTLA-4	14D3	3161004
^{162}Dy	CD69	FN50	3162001
^{163}Dy	CD56	NCAM16.2	3163007
^{164}Dy	CD95/Fas	DX2	3164008
^{165}Ho	CD45RO	UCHL1	3165011
^{167}Er	CD27	O323	3167002
^{168}Er	CD154/CD40L	24-31	3168006
^{169}Tm	CD366/TIM-3	F38-2E2	3169028
^{170}Er	CD3	UCHT1	3170001
^{171}Yb	CD185/CXCR5	RF8B2	3171014
^{172}Yb	CD57	HCD57	3172009
^{174}Yb	HLA-DR	L243	3174001
^{175}Lu	CD223/LAG-3	11C3C65	3175033
^{176}Yb	CD127/IL-7Ra	A019D5	3176004
^{209}Bi	CD137/4-1BB	4B4-1	3209015

Table S2. First antibody panel for flow cytometry measurement. PBMC from the full cohort including controls, patients, household members, hospital healthcare workers, general practitioners, and pre-COVID donors were analyzed.

Antibody	Clone	Fluorochrome	Supplier	Catalogue Number
CD45	REA747	VioGreen	Miltenyi Biotec	130-110-638
CD3	REA613	VioBright R720	Miltenyi Biotec	130-127-377
CD4	RPA-T4	PE-Cy5.5	Invitrogen	35-0049-42
CD8	REA734	PE-Vio770	Miltenyi Biotec	130-110-680
CD27	O323	BV570	BioLegend	302825
CD28	CD28.2	BV711	BD Biosciences	563131
CD38	REA572	VioBright V423	Miltenyi Biotec	130-127-178
CD45RA	HI100	BV786	BD Biosciences	563870
CD69	FN50	BB700	BD Biosciences	747520
CD95/Fas	REA738	PE-Vio615	Miltenyi Biotec	130-113-009
CD134/OX40	ACT35	PE-Cy5	BD Biosciences	551500
CD137/4-1BB	4B4-1	BV605	BD Biosciences	745256
CD154/CD40L	REA238	VioBright B515	Miltenyi Biotec	130-122-800
CD197/CCR7	REA108	APC	Miltenyi Biotec	130-120-460
CD223/LAG-3	REA351	PE	Miltenyi Biotec	130-120-470
TIGIT	741182	BV650	BD Biosciences	747840
Fixable Viability Stain	--	Near IR	Invitrogen	L34976

Table S3. Second antibody panel for flow cytometry measurement. PBMC from controls, patients, and household members were analyzed as supplementary to the first panel.

Antibody	Clone	Fluorochrome	Supplier	Catalogue Number
CD45	REA747	VioGreen	Miltenyi Biotec	130-110-638
CD3	SK7	AF700	BioLegend	344821
CD4	SK3	PE	BD Biosciences	345769
CD8	REA734	PE-Vio770	Miltenyi Biotec	130-110-680
CD279/PD-1	EH12.2H7	BV421	BioLegend	329919
HLA-DR	L243	BV785	BioLegend	307641
CD366/TIM-3	F38-2E2	PE-Dazzle 594	BioLegend	345033
CD152/CTLA-4	BNI3	AF647	BioLegend	369625
CD69	REA824	APC-Vio770	Miltenyi Biotec	130-112-805
CD137/4-1BB	4B4-1	BV605	BD Biosciences	745256
CD134/OX40	ACT35	PE-Cy5	BD Biosciences	551500
CD154/CD40L	REA238	VioBright B515	Miltenyi Biotec	130-122-800
Fixable Viability Stain	--	V575	BD Biosciences	565694

Table S4. List of recovered patients with "long COVID" symptoms. There were 79 recovered patients (out of 166 in the full cohort) experiencing long COVID, 40 of which were initially classified as mildly ill patients, 28 were moderately ill, and 11 were severely ill.

Table S5. Demographic and clinical details of all participants. Participants in this study were divided into these categories: (i) recovered COVID-19 patients (n = 168), (ii) household members of the recovered patients (n = 27), (iii) controls (n = 259), (iv) general practitioners (n = 37), (v) hospital healthcare workers (n = 91), and pre-COVID donors (n = 24).

Table S6. List of samples analyzed by mass cytometry. PBMC from 45 donors, including 30 patients and 15 pre-COVID donors, were used in mass cytometry analysis.

Table S7. List of custom-synthesized MHC specific SARS-CoV-2 peptides. These peptides were based on a curated list of known SARS-CoV-2 epitopes, extracted from various existing studies using ELISpot, in silico prioritization, and TCR-epitope simulation.

Table S8. List of annotated TCR–epitope specificity. TCR-seq was performed on enriched $CD8^+$ T-cells. Epitope specificity of the TCRs was predicted with TCRex. Clustering of TCRs with similar CDR3 sequences was performed using ClusTCR.

Table S9. List of HLA-A02 serotype and annotated TCR–HLA specificity. HLA-A02 serotype was obtained by flow cytometry. TCR–HLA specificity was annotated using computational simulation. Among the 36 patients with available HLA typing and experimental data on which MHC molecules can present recognized epitopes, 23 were HLA-A02 positive, and 13 were HLA-A02 negative. All HLA-$A02^+$ patients were found to recognize at least one epitope known to be presented by HLA-A02. At least one HLA-A02 restricted epitope was recognized by 9 out of 13 (69%) HLA-$A02^-$ patients and 18 out of 23 (78%) HLA-$A02^+$ patients, with the

HLA-A02$^+$ patients having a significantly higher fraction of recognized HLA-A02 restricted epitopes overall (MU $p = 0.029$).
